# Trends in brain MRI and CP association using deep learning

**DOI:** 10.1007/s11547-024-01893-w

**Published:** 2024-10-10

**Authors:** Muhammad Hassan, Jieqiong Lin, Ahmad Ameen Fateh, Yijiang Zhuang, Guisen Lin, Dawar Khan, Adam A. Q. Mohammed, Hongwu Zeng

**Affiliations:** 1 Department of Radiology, Shenzhen Children’s Hospital, Futian, Shenzhen, 518038 Guangdong China; 2 King Abdullah University of Science and Technology, Thuwal, 6900 Kingdom of Saudi Arabia; 3 School of Computer Science and Engineering, Southeast University, Nanjing, 211189 China

**Keywords:** Cerebral palsy, Early intervention, MRI coupling, Vulnerabilities, Deep learning

## Abstract

Cerebral palsy (CP) is a neurological disorder that dissipates body posture and impairs motor functions. It may lead to an intellectual disability and affect the quality of life. Early intervention is critical and challenging due to the uncooperative body movements of children, potential infant recovery, a lack of a single vision modality, and no specific contrast or slice-range selection and association. Early and timely CP identification and vulnerable brain MRI scan associations facilitate medications, supportive care, physical therapy, rehabilitation, and surgical interventions to alleviate symptoms and improve motor functions. The literature studies are limited in selecting appropriate contrast and utilizing contrastive coupling in CP investigation. After numerous experiments, we introduce deep learning models, namely SSeq-DL and SMS-DL, correspondingly trained on single-sequence and multiple brain MRIs. The introduced models are tailored with specialized attention mechanisms to learn susceptible brain trends associated with CP along the MRI slices, specialized parallel computing, and fusions at distinct network layer positions to significantly identify CP. The study successfully experimented with the appropriateness of single and coupled MRI scans, highlighting sensitive slices along the depth, model robustness, fusion of contrastive details at distinct levels, and capturing vulnerabilities. The findings of the SSeq-DL and SMSeq-DL models report lesion-vulnerable regions and covered slices trending in age range to assist radiologists in early rehabilitation.

**Supplementary Information:**

The online version contains supplementary material available at 10.1007/s11547-024-01893-w.

## Purpose

Cerebral palsy (CP) is a neurological disorder affecting the cerebral cortex’s brain regions and associated with motor functions, including movement, balance, and posture [24]. It is the most prevalent motor disability in children, resulting from abnormal brain development and affecting a person’s ability to control their muscles. The CP complication can be caused by genetic and environmental factors [15]. Along with problems related to movement and posture, CP patients may also experience seizures, spine changes, joint issues, intellectual disability, and problems with vision [12]. CP developing was reported in between 3 and 10 out of 1000 children [15]. CP is not treatable and irreversible, but early identification using cutting-edge technologies can help with medications and supportive treatments [17, 27]. The ideal treatment age for CP is 1 to 24 months, but it is challenging owing to uncooperative younger age [16]. Early diagnosis is essential as it mitigates lifelong issues [28]. However, the detection of finding early biomarkers for CP is challenging because of the potential recovery of infants and gross motor functions [4, 9]. Every child with CP has a unique composition of neurological symptoms that shape their functional profile [28]. Brain imaging can be practical to visualize such composition and beneficial in identifying CP occurrence, implications for treatment, and diagnosis [11]. An intelligent system can inevitably capture such composition from their brain’s functional and structural visuals.

The prediction of CP has been carried out from a variety of dataset modalities, including spontaneous general movement [4, 18], naked eye [10] and body movement [21], gait patterns [29], DNA [4], and neuroimaging [1, 11, 19, 25], including CP association [11], classification [19, 31], investigation [32], and early diagnosis [9, 18, 25]. It is challenging to accurately identify CP through physical movement disorders’ visuals, intellectual disabilities, and other manifestations [25]. Magnetic resonance imaging (MRI) has become the most widely used imaging modality for diagnosing neurological disorders [25]. The MRI sequences provide insights into the pathological factors associated with brain structural findings in the newborn brain [26]. The majority of CP children have been examined via lesion outlining [11] and classifying CP using brain MRI [19]. Therefore, brain MRI visuals may accurately depict distinct patterns in brain regions associated with CP [34].

In MRI brain visuals, selecting the appropriate contrast(s) for a given pathology remains challenging. Moreover, the joint adventure of contrastive scans can lead to better representations of brain structure and abundant complementary information useful for accurate CP identification [8, 30]. Therefore, multi-contrast or sequence (MS-MRI) provides abundant complementary information reflecting the characteristics of the internal tissues to disentangle the pathological characteristics of neurological disorders [8]. The shared information between inter-contrastive images can benefit learning lesions and vulnerable brain regions [30]. Nonetheless, the selection of the most informative and appropriate MR (coupling) contrasts is pivotal. However, CP identification via brain MRI may lead to misclassification results using visual inspection, early age factors, dearth single modality, and limited automatic methods to capture minute and lesion sensitive regions [35–37]. The significant rise of misclassification by visual inspection, early age factors, and the dearth of singleton modality demand robust and efficient DL models. The DL models learn infinitesimal information from MRI, whereas clinicians may not be able to observe these minute and imperceptible lesions via naked-eye examination.

Automatic and data-driven learning methods-based attempts have been undertaken to identify and classify CP with various network structures [29]. DL-based strategies have recently shown promising performance in a wide range of tasks, particularly in medical imaging [13], identifying and classifying neurological disorders [3, 14], reconstruction [2], and prognosis [20]. Some studies [7] have utilized infants’ spontaneous movement from videos to identify CP and distinguish normal and abnormal body movements [21]. Most of the studies conducted the CP identification and classification using the apparent motor actions of subjects [14]. The diagnostic accuracy of CP patients in clinical trials using DL learning reached 88.6% [25]. The study [5] identified factors associated with autism spectrum disorder in adolescents with CP using AI. However, most existing attempts for the classification result in a limited age ranging from 2 to 6 years, while poor results were observed for the early age [4, 18]. The excessive anatomical variations in whole brain mapping caused by the infants’ body uncooperative movements make CP identification more challenging [6]. It is evident from the literature that the classification of CP from MRI is challenging and requires investigations on the anatomical details [6]. The literature studies are limited to classifying CP employing attention mechanisms for lesion-vulnerable regions either on SI-MRI or MI-MRI [18]. However, the study [33] has attempted to predict CP using a channel attention module from infants’ body movements with 91.67% accuracy rather than MRI. Furthermore, the study [38] utilized Cascaded LeNet as backbone architecture to classify CP into subtypes with the segmented of specific brain regions, however, lacking of CP vulnerable regions from the model trained weights. Most of the literature studies predict CP from physical body movements, poses, and gestures where limited DL architectures are proposed for CP identification from brain MRIs with embedding attention mechanisms to outline vulnerable brain regions between HC and CP. In this study, the proposed DL architectures identify CP from brain visuals, selecting an optimal single-sequence (SS-MRI) and coupling of MRI (MS-MRI) sequences, utilizing complementary information via parallel computing, and learning CP vulnerable regions using specialized attention mechanisms. The introduced specialized DL models (SSeq-DL and SMSeq-DL) predict early and accurately, setting a benchmarking study in terms of optimal SS-MRI and MS-MRI and reporting CP-associated regions. The main contributions are:

- Introduction of specialized DL models training on SSeq-MRI and MS-MRI.
- To optimally select SS-MRI for timely intervention in CP identification from infancy to adolescence.
- Benchmarking of MS-MRI coupling to reduce CP misclassification employing contrastive learning.
- Visualizing CP’s vulnerable regions in MRI scans and slices to assist radiomics.
- Delving into the trends associated with HC and CP from infancy to adolescence.

## Material and methods

Two major studies are carried out: (1) modeling network architecture appropriate for CP identification via SS-MRI and (2) utilizing parallel computing for MS-MRI that runs in the pseudocode (Algorithm 1). Lines 1 to 6 select network parameters, input, and combining visuals, as well as the introduced architectures. The first loop run for single-sequence-DL (SSeq-DL) via SS-MRI is for CP identification. The second loop covers the training and evaluation of single-to-multi-sequence DL (SMSeq-DL) over MS-MRI. The output of the pseudocode (Algorithm 1) is the optimal selected SS-MRI and MS-MRI for CP.

**Algorithm 1:**
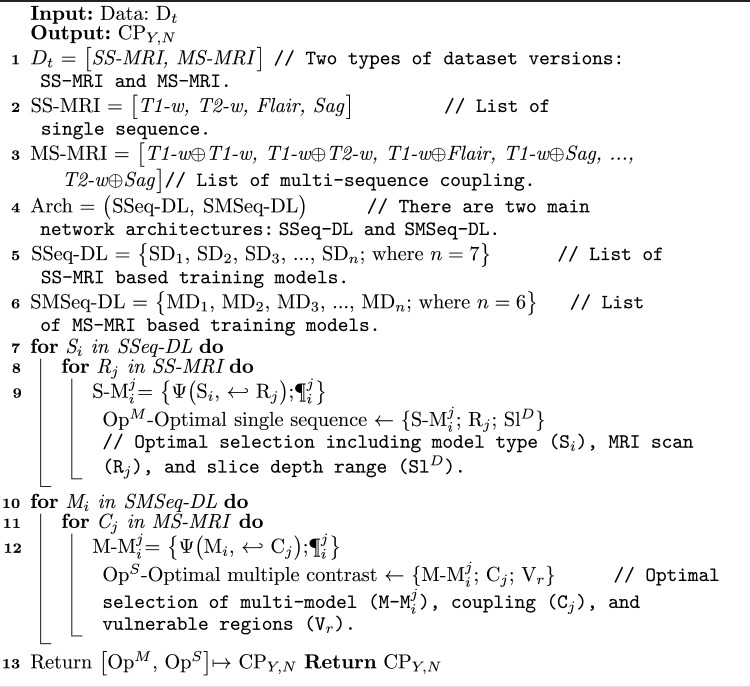
Experimental and training pseudocode

The generalized network structure for SS-MRI and MS-MRI is formulated as follows:

1 $$\begin{aligned} SMS = \Psi \Big [ \Upsilon \big (SC/MC \big ),~\Rightarrow ,~ \Re \{SC/MC,~\P \big (\omega , \beta \big )\} \Big ] \end{aligned}$$

where $\Psi$ is the end-to-end learning composed of preprocessing $\Upsilon$ followed by network learning $\Re$. The preprocessing $\Upsilon$ employs single-scan (SC-MRI) or multi-sequence (MS-MRI) input MR visuals to make a robust and reliable DL model. The preprocessed MRI scans are then passed to a neural network $\Re$, which learns weights $\omega$ and biases $\beta$ toward CP identification.

### Single scan MRI-based modeling

Numerous experiments are held to select the appropriate scan and architecture for CP prediction (Supplementary Figure 1 and Table 3). SSeq-DL is the efficient architecture among the competitor models, which is formulated as follows:

2 $$\begin{aligned} {SSeq-DL} = \sum _{i}^{j}MC_{i} \{ MR_s:~\Rightarrow ~(\Upsilon \vDash A.M)~||~(\Upsilon \Vdash A.M) \oplus ||\otimes ,~E||M||L \}. \end{aligned}$$

**Fig. 1:**
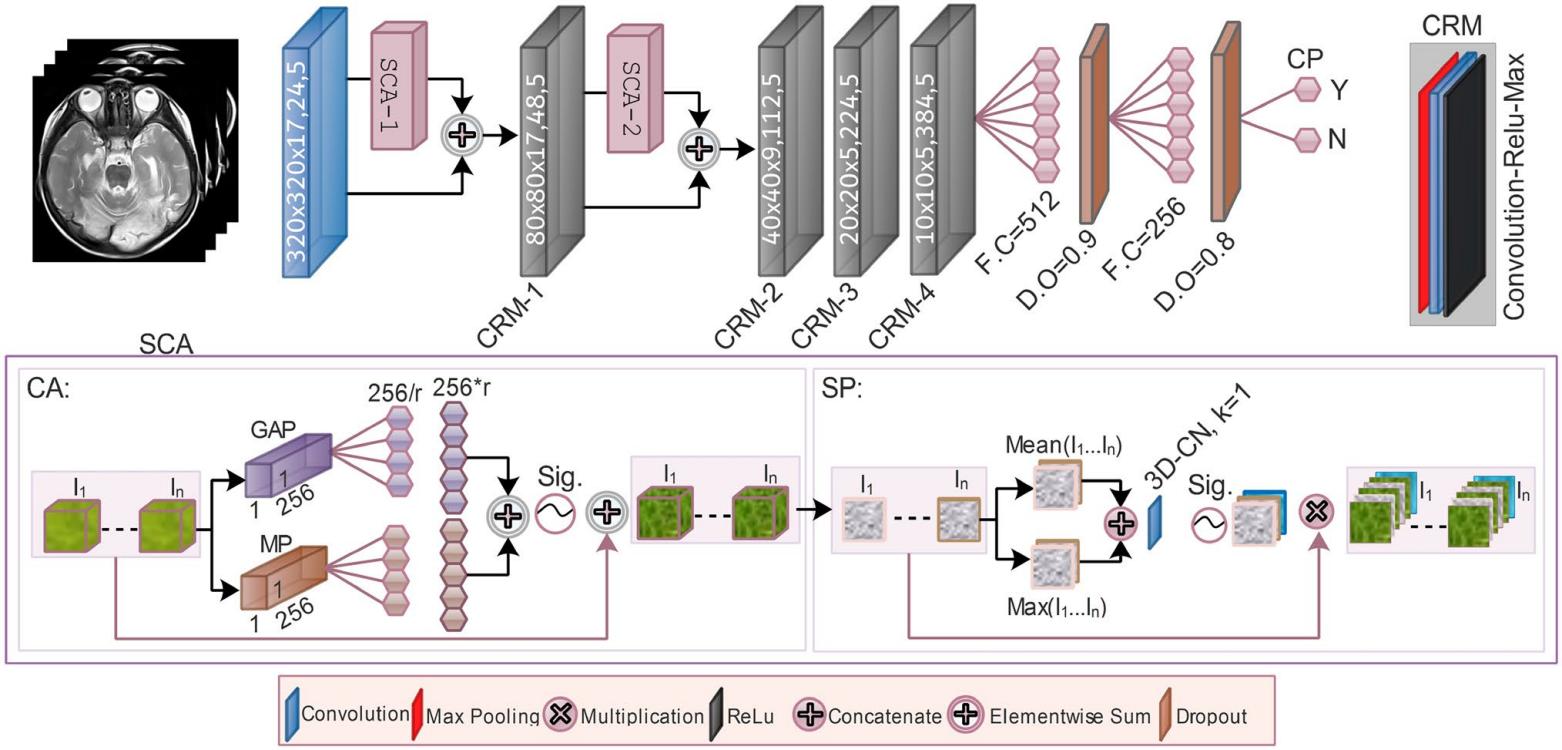
Architecture of single-sequence (SS)-based DL (SSeq-DL) model

The DL trains for all the samples ${i\rightarrow k}$ and applies the $\Upsilon$ to MR images. Attention mechanism (AM) is used in parallel $\vDash$ or sequentially $\Vdash$. The parallel outcomes, either received from residual connections or through AM, are element-wise summed $\oplus$ and multiplied $\otimes$. Different fusion levels, early *E*, mid *M*, or late level *L*, are performance-based elaborated. SSeq-DL is the effective SS-MRI-based model, receiving SS-MRI as input, with optimal placement of both spatial (SA) and channel (CA) attention (SCA) (Fig. 1). The SCA is placed twice in SSeq-DL, depending on the performance. There are four convolution-rely-max (CRM 1-4) blocks, followed by fully connected (FC) and dropout layers to predict CP.

### Multi-contrast learning and CP identification

**Fig. 2:**
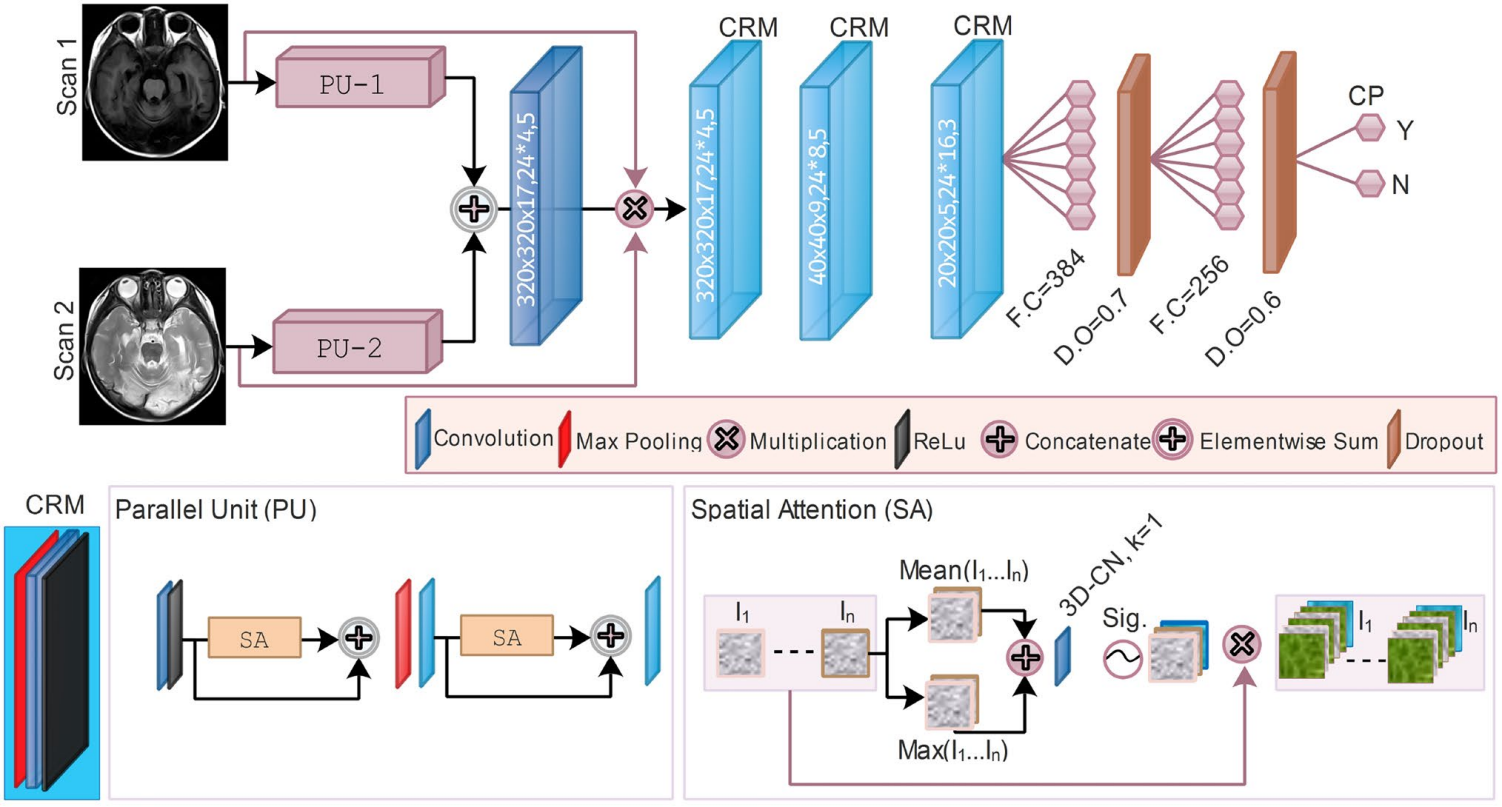
Network architecture of single-to-multi-sequence DL (SMSeq-DL)

The involvement of contrastive learning aims to improve prediction accuracy. The parallel computing or partial Siamese learning units receive MS-MRI (Fig. 2 and Supplementary Figure 2). The resemblance or disparity features in the Siamese network have a pivotal role in CP prediction. The partial Siamese fuses the parallel units at mid-level to allow the network to comprehend prior and post-fusions. The model retains the weights of each input scan to the mid-level, then shares the common weights, and utilizes discrepant features to enhance robustness in class imbalance. Thus, this study benchmarks against the competitors, including SSeq-DL and state of the art (SOTA) (see details in Supplementary). SA-and-CA attentions are employed with variant architectures to emphasize the exciting features associated with CP (Supplementary Figure 2).

SMSeq-DL receives MS-MRI $MR_m$ for a single subject and learns CP-related patterns motivated by contrastive information formulated as follows:

3 $$\begin{aligned} SMS-DL = \sum _{i}^{j}MC_{i} \{ MR_m:~\Rightarrow ~\Upsilon \rightarrow (Ni_p,\oplus ||\otimes ,~Ni_{p+1})~\prec \ell _d(Ni_p\odot Ni_{p+1}), AM, \omega , \beta \}, \end{aligned}$$

The contrastive learning *MC* occurs from MS-MRI in the range of $i\rightarrow j$. The preprocessing $\Upsilon$ versions of $MR_m$ pass to the partially parallel units, such as $Ni_p$ and $Ni_{p+1}$, and merge either with element-wise sum $\oplus$ or multiplication $\otimes$. The merging operation $\prec$ carries at different depth levels $\ell _d$ and proceeds to a joint $(Ni_p\odot Ni_{p+1})$ feature representations. Similarly, each network may apply attention mechanisms (AM) at different positions depending on the network’s learned weights $\omega$ and biases $\beta$. SMSeq-DL is composed of a Conv-Layer, CA, SA, sequential (Seq), parallel (Paral), max-pool (MP), and FC (Fig. 2, Supplementary Figure 2 and Table 4). SMSeq-DL has a parallel unit (PU) with each SA one after another, middling with MP and Conv-Layer (Fig. 2).

### Dataset collection

There are 716 subjects; 327 are patients, and 389 are controls. The age-wise distribution into eight groups is tabulated in Table 2 (first row). Each subject has four MRI sequences collected. The control participants’ MRIs were obtained using Skyra, GE, and Phillips machines, with ratios of 123, 138, and 128, respectively. However, most patients’ MRIs were obtained using GE (282), while a minority of patients’ MRIs were recorded using Skyra (44). The table below (Table 1) provides the statistical distribution of the acquired MRI data. The magnetic field strengths 1.5(T) and 3(T) influence the training and testing results of deep learning models in various aspects. DL models benefit from the increased resolution and contrast of 3T MRI in learning small anatomical structures in the brain [39–41]. However, diverse training datasets, including images from 1.5T and 3T MRI scanners, can help develop more robust DL models training [42] (Supplementary Table 6). The proposed models’ training is equipped with dynamic augmentation to overcome the challenging low signal intensity factor (see more details in Supplementary section).

**Table 1:** Skyra, GE, and Phillips MRI scanners-based dataset collection and comparison

MRI machine	HC	Patient	PSNR	Contrast between tissues	Information potential	Anatomical details
Skyra (3T)	123	44	High	High	High	High
GE (1.5)	138	282	Low	Low	Low	Low
Philips (3T)	128	1	High	High	High	High
Total	389	327				

The inclusion and exclusion criteria are illustrated in Table 2; see further details in Supplementary Section 0.4.

**Table 2:** The age-wise sample distributions for health controls and patients into eight groups followed by the inclusion and exclusion criteria

Subject	G_1	G1_3	G3_5	G5_8	G8_11	G11_13	G13_15	G15_18
Age range	<1	>1:$<=$2	>2:$<=$4	>4:$<=$7	>7:$<=$10	<10:$<=$13	>13:$<=$15	> 15
Controls	16	68	59	101	82	49	10	3
CP	52	95	90	54	13	10	3	6

Subject	Criteria	Description						
CP	Inclusion	1. CP confirmation via international consensus criteria.						
		2. Complete routine MRI examination.						
	Exclusion	1. Having other neurological disorders, traumatic brain injuries, and systemic illnesses.						
		2. Artifacts and poor image quality.						
Controls	Inclusion	1. Apgar score >=8 at 1 min and 7 min after birth. Full term and of normal weight.						
		2. No abnormality.						
	Exclusion	1. Perinatal asphyxia, intrauterine distress, or any neurological disease, traumatic brain injuries, or systemic illnesses.						
		2. Artifacts and poor image quality.						

The dataset is acquired from children in the pediatric rehabilitation center, and the MRIs are diagnosed and classified as either CP or HC in the stationed hospital. To train DL models, the DICOM MRIs were converted to NifTi (.nii) format using a cross-platform image viewer (MRIcron) [43]. The NifTi-formatted MRIs were loaded and converted into a NumPy structure representation using the Python function (Scikit). The conversion process has been carried out under the supervision of radiologists in the Radiology department of the stationed Children’s Hospital, while visualizations were demonstrated of distinct brain regions spread over a range of slices [43]. The images were then transformed and aligned to have the same dimensions and depth using Scikit.

### Clinical characteristics of patients

The study recruited HC and CP children from the stationed hospital. The CP children are diagnosed with CP disorder, and most of them are inpatients. However, HC is mostly outpatient and is examined as healthy in the stationed children’s hospital. The clinical characteristics of patients primarily revolve around individuals diagnosed with cerebral palsy, highlighting various aspects in terms of clinical characteristics. The clinical characteristics of patients diagnosed with CP confined to CP types (e.g., 1 = spastic; 2 = dyskinetic; 3 = ataxic; 4 = hypotonic; 5 = mixed; and 6 = rigid), birth details (e.g., weight, preterm or full term, gestational age), mode of delivery, and specific perinatal risk factors like respiratory distress, gestational diabetes, or instances of hypoxic asphyxia at birth (Table 3). It also details the familial medical history, the presence of movement disorders, mental retardation, and whether there were issues like swallowing dysfunction, speech disorders, or epilepsy. Treatments and interventions noted include standard rehabilitation treatments, surgical procedures, motor delay training, balance function training, advanced therapies such as botulinum toxin injections, hyperbaric oxygen therapy, and cord blood transplantation treatments, indicating a broad, multifaceted approach to managing cerebral palsy. Each patient’s record, although varying in the amount of detail and specific conditions, collectively underscores the complexity of cerebral palsy care, reflecting individualized treatment plans that address both the neurological and physical aspects of the diagnosis. In a nutshell, the key clinical characteristics besides demographics include CP types, paralysis location, birth data, perinatal risk factors, treatment and intervention, neurodevelopmental and functional status, and rehabilitation and therapy.

**Table 3:** Demographic and clinical characteristics of HC and CP subjects

Characteristics	CP	HC	CP and HC	*P* value
Age: Mean [s.d]	3.446 [3.227]	6.280 [3.781]	4.863 [3.811]	<0.001***
Gender: N [Male/Female]	327 [206/121]	381 [233/148]	–	–
Weight: Mean [s.d]	7.523 [8.335]	14.365 [13.970]	5.628 [13.582]	<0.001***
CP Type				
CP (not classified): N [%]	98 [29.96%]	–	–	–
Spastic: N [%]	192 [58.71%]	–	–	–
Dyskinetic: N [%]	18 [5.50%]	–	–	–
Ataxic: N [%]	0 [0%]	–	–	–
Hypotonic: N [%]	0 [0%]	–	–	–
Mixed: N [%]	19 [5.81%]	–	–	–
Rigid: N [%]	0 [0%]	–	–	–
Paralysis Type	–	–	–	–
Mixed/Unknown: N [%]	127 [38.83%]	–	–	–
Monoplegia: N [%]	3 [0.91]	–	–	–
Diplegia: N [%]	59 [18.04%]	–	–	–
Triplegia: N [%]	0 [0%]	–	–	–
Hemiplegia: N [%]	62 [18.96%]	–	–	–
Tetraplegia: N [%]	76 [23.24%]	–	–	–
CP term: Preterm N [%], Full term N [%]	155 [47.40%], 172 [52.59%]	–	–	–
Delivery: Cesarean N [%], Normal [%]	146 [44.64%], 181 [55.35%]	–	–	–
Movement Disorder: Yes N [%], No N [%]	274 [83.79%], 53 [16.20%]	–	–	–
Mental retardation: Yes N [%], No N [%]	156 [47.70%], 171 [52.29%]	–	–	–
Swallowing dysfunction: Yes N [%], No N [%]	42 [12.84%], 285 [87.15%]	–	–	–
Speech disorder: Yes N [%], No N [%]	114 [34.86%], 213 [65.13%]	–	–	–
Epilepsy: Yes N [%], No N [%]	97 [29.66%], 230 [70.33%]	–	–	–
Rehabilitation received: Yes N [%], No N [%]	253 [77.37%], 74 [22.62%]	–	–	–
Motor delay function: Yes N [%], No N [%]	267 [81.65%], 60 [18.34%]	–	–	–
Traction: Yes N [%], No N [%]	5 [1.52%], 322 [98.47%]	–	–	–
Balance function training: Yes N [%], No N [%]	8 [2.44%], 319 [97.55%]	–	–	–
Hyperbaric oxygen chamber therapy: Yes N [%], No N [%]	52 [15.90%], 275 [84.09%]	–	–	–
Direct current therapy: Yes N [%], No N [%]	114 [34.86%], 213 [65.13%]	–	–	–
Botulinum toxin injection: Yes N [%], No N [%]	26 [7.95%], 301 [92.04%]	–	–	–
Wearing aligner: Yes N [%], No N [%]	22 [6.72%], 305 [93.27%]	–	–	–
Cord blood transplantation treatment: Yes N [%], No N [%]	14 [4.28%], 313 [95.71%]	–	–	–

***$=p<$0.001, **$=p<$0.01, *$=p<$0.05, $N=$Samples, $\%=$Percentage, s.d=Standard deviation

### MRI slices distribution and covered brain regions

**Fig. 3:**
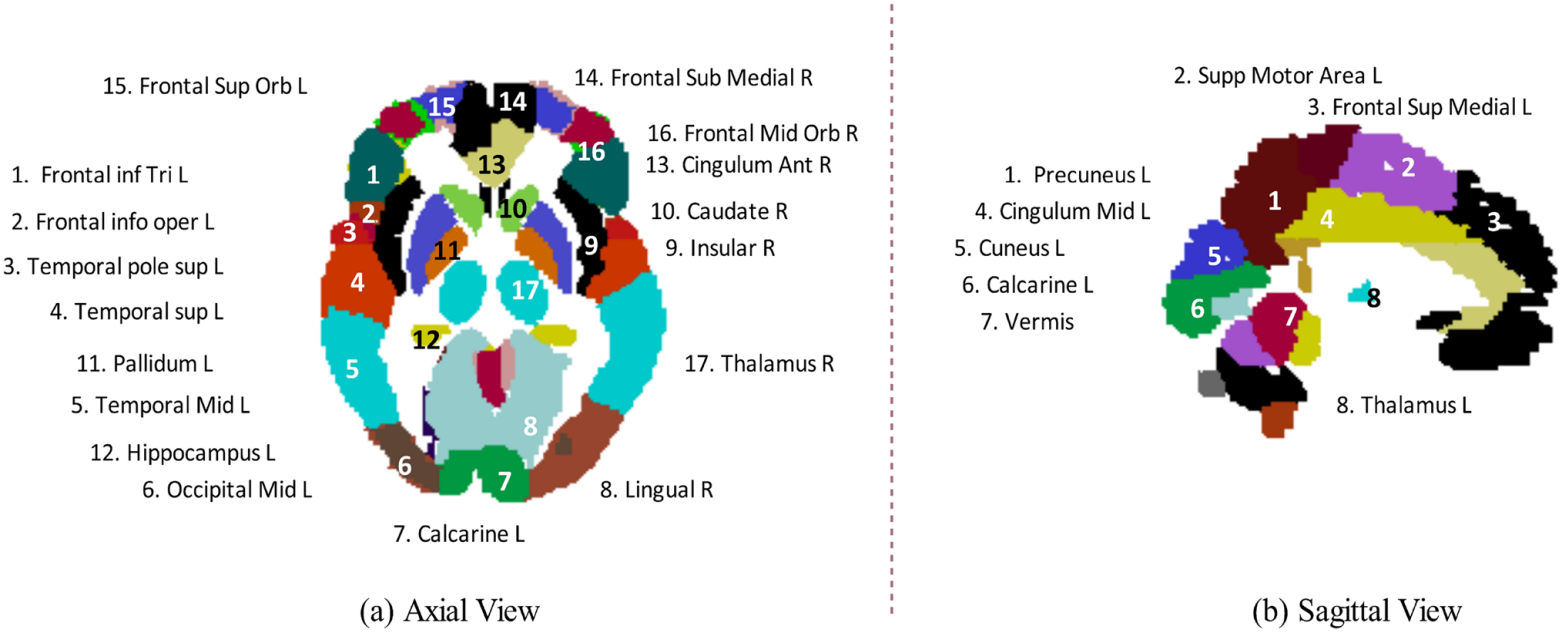
Demonstration of axial and sagittal views of brain MRI. Different regions are labeled according to their viewed positions

The dataset used in this investigation included T1-w, T1-sag, T2-w, and Flair sequences obtained from axial or sagittal views. The brain MRI’s axial view may reveal distinct brain tissues along slice depth, which are annotated and shown in Fig. 3a. There are region variations along the slices; however, a single slice with annotations is shown in Fig. 3a for understanding. The visualizations are made using the MIcron software tool to demonstrate distinct brain regions spread over a range of slices [43]. Furthermore, we also outline the sagittal view of brain MRI with covered regions (Fig. 3b). Highlighting the regions of interest aims to utilize the analysis and evaluation results of the regions of interest that are focused on the attention mechanisms of DL models and associated with CP neurological disorders. The details of the axial and sagittal view region distributions of the collected datasets are described in Supplementary section. Furthermore, seventeen slices are distributed into four groups, where groups 1–4 include slices 1–4, 5–8, 9–13, and 13–17. All the groups together with covered regions are tabulated in Supplementary section (Tables S-1 and S-2). Well-trained radiologists and neuroradiologists annotated the collected dataset, including labeling poor-quality images, analyzing the results, and evaluating the samples.

## Results

### Single scan (SC) brain MRI and CP association

**Fig. 4:**
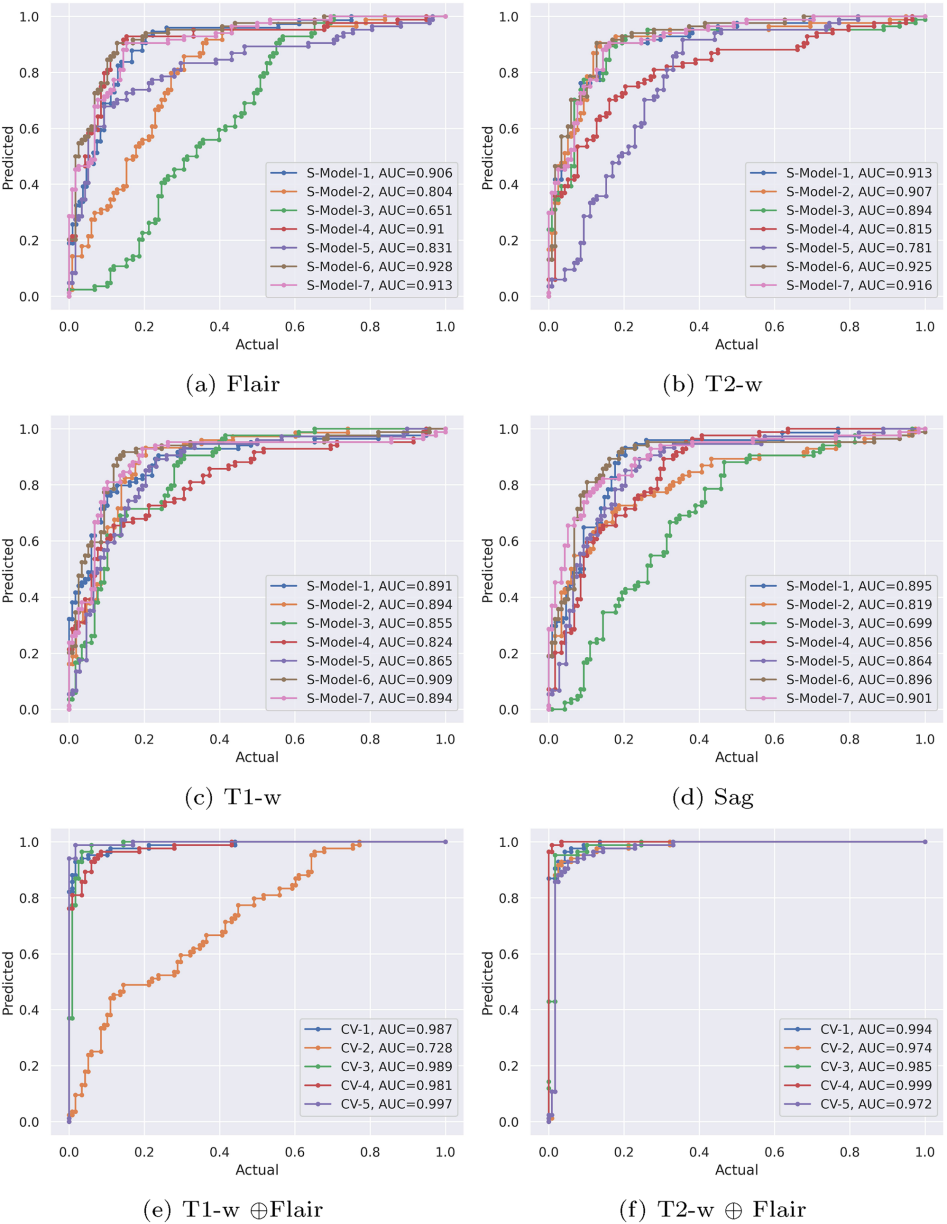
Seven DL models train on, **a** Flair, **b** T2-w, **c** T1-w, and **d** Sag scans. The AUCs for coupling T1-w $\oplus$ Flair **e** and T2-w $\oplus$ Flair **f** with fivefold cross-validations are shown

The experiments in this study aim to discover an optimal SS-MRI-based DL architecture and an appropriate SS-MRI for CP association. The underlying seven DL architectures’ trained results as AUC curves are plotted in Fig. 4a–d. Among these, T2-w was found to have a higher cumulative AUC score compared to the counterpart T1-w, Sag, and Flair. The suitability of the T2-w scan for CP prediction is further elaborated and verified using confusion metrics with accuracies ranging from 87.25% to 90.19% (Supplementary Table 7). On the contrary, the Sag scan was observed to have poor performance. In network architecture-wise optimal selection, Model-6’s (SSeq-DL) performance was robust over SS-MRI scans. It can be deduced from the statistical results that Model-6 (SSeq-DL) is more appropriate while training on SC scans (88.23% using Sag to 89.21% using T2-w). A few of the models, including Model-3 (89.21% using T2-w), Model-4 (90.19% using T2-w), and Model-7 (90.19%, 89.19%, and 88.23% using T2-w, Flair, and Sag), show slightly better performance; however, poor performance is seen for the rest of the scans (Flair, T1-w, and Sag). Thus, the network structure of Model-6 is employed for CP identification and named SSeq-DL in this study.

The cumulative prediction accuracies for SS-MRI are illustrated in Table 4. From the training statistics (Table 4), T2-w and Flair’s scans were reported with significance regarding CP prediction. A minor lower CP prediction cumulative results are reported for T1-w and Sag.

**Table 4:** The SS-MRI-based CP prediction using SSeq-DL is shown

Scan	Model	TP	FP	TN	FN	Specificity	Sensitivity	PPV	NPV	F$_{1}$	Accuracy (%)
T2-w	SSeq-DL	56	4	35	7	0.8974	0.8889	0.9334	0.8334	0.8931	89.21
Flair	SSeq-DL	55	5	36	6	0.8780	0.9016	0.9166	0.8571	0.8896	89.21
T1-w	SSeq-DL	53	7	37	5	0.8409	0.9137	0.8833	0.8809	0.8758	88.23
Sag	SSeq-DL	53	7	37	5	0.8409	0.9137	0.8833	0.8809	0.8758	88.23

### Enhancing CP identification using MS-MRI-based learning

There are the proposed multi-contrastive-based models with six architecture versions (Supplementary Table 4) using the coupling of MS-MRI. The architecture of SMSeq-DL (Fig. 2) was found to have efficient performances among the six underlying architectures (Table 4). In scan-wise coupling, the fusion of T1-w $\oplus$ Flair and T2-w $\oplus$ Flair showed significant results for CP prediction (Fig. 4e–f). To further evaluate the robustness based on the coupling, the fivefold cross-validations of T1-w $\oplus$ Flair and T2-w $\oplus$ Flair are shown in Table 5L. The cumulative accuracies for coupling T1-w $\oplus$ Flair and T2-w $\oplus$ Flair received 94.70% and 94.31% scores, respectively. The misclassification of CP from MRI in clinical practices can be reduced to a low level by employing contrastive information learned from the coupling of MS-MRI couplings. However, the joint adventure of sagittal and other MRI scans resulted in poor performance (Supplementary Table 10). Overall, MS-MRI-based learning outperformed SC-MRI-based learning.

The drawn AUC curves for the SMSeq-DL where the coupling of T2-w $\oplus$ Flair (Fig. 4e-d) is shown with robust results (see details in Supplementary Figure 4). The confusion matrix as an evaluation metric is significant. The AUC curves and confusion matrix show the robustness of the proposed SMSeq-DL over T1-w $\oplus$ Flair and T2-w $\oplus$ Flair.

**Table 5:** The illustration of the fivefold cross-validations of MS-MRI includes T1-w $\oplus$ Flair and T2-w $\oplus$ Flair. The evaluation metrics have specificity, sensitivity, and accuracy. The tabulated information is excerpted from significant results from Supplementary Table 10

Fused Models	Model	TP	FP	TN	FN	Specificity	Sensitivity	PPV	NPV	F$_1$	Acc
	CrossValidation-1	57	3	38	4	0.926	0.9344	0.9500	0.9047	0.9306	93.13
	CrossValidation-2	56	4	38	4	0.9047	0.9333	0.9333	0.9047	0.9188	92.15
Fusion of T1-w and Flair	CrossValidation-3	57	3	41	1	0.9318	0.9827	0.9500	0.9761	0.9566	96.07
	CrossValidation-4	57	3	40	2	0.9302	0.9661	0.9500	0.9523	0.9478	95.09
	CrossValidation-5	58	2	41	1	0.9534	0.9830	0.9666	0.9761	0.9680	97.05
	Cumulative scores					0.9294	0.9599	0.9500	0.9428	0.9443	94.70
	CrossValidation-1	57	3	40	2	0.93023	0.96610	0.9500	0.9723	0.9478	95.09
	CrossValidation-2	56	4	38	4	0.9047	0.9333	0.9333	0.9523	0.9047	92.15
Fusion of T2-w and Flair	CrossValidation-3	55	5	40	2	0.8936	1	0.9166	1	0.9438	95.09
	CrossValidation-4	59	1	40	2	0.9756	0.9642	0.9833	0.9523	0.9797	98.03
	CrossValidation-5	54	6	40	2	0.8695	0.9642	0.9000	0.9523	0.9144	92.15
	Cumulative scores					0.9147	0.9661	0.9366	0.9523	0.9392	94.31

Positive predictive value (PPV), negative predictive value (NPV)

### Brain MRI vulnerabilities along slices

#### Complementary information fusion and CP identification

**Fig. 5:**
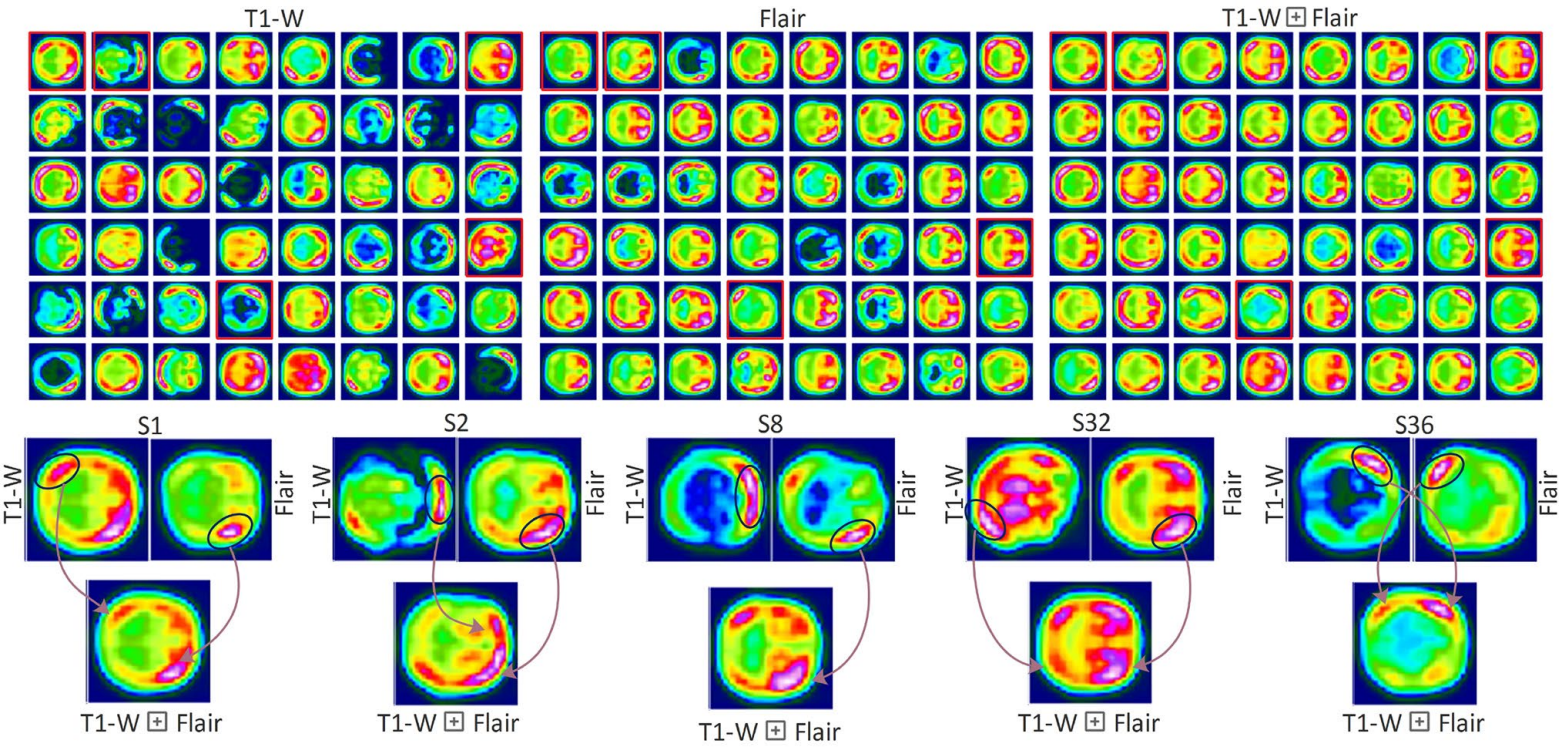
Visualization of a sample ( T1-w $\oplus$ Flair) from two fusion modalities. The first row depicts visuals for T1-w and Flair and their fusion ($\oplus$). A few random slices, including S1, S2, S8, S32, and S36, are shown in second row

To circumvent the question raised about improvement in CP identification by using MS-MRI, we aim to visualize the learned features as depicted in Fig. 5. Among the coupling, only T1-w $\oplus$ Flair is used for illustration purposes (see Supplementary for T2-w $\oplus$ Flair). The visualization aims to interpret the CP-associated vulnerable brain regions. Figure 5 shows the feature maps for the receiving input T1-w (First column) and Flair (Second column) following by the fusion of complementary information as fusion (Third column). The visualized results are the cumulative mean of the network weight corresponding to T1-w, Flair, and T1-w$\oplus$Flair. Two MRI scans’ representations feed to the parallel network unit, which is visualized in Fig. 5. There are 48 feature maps at the chosen network level. The filters in parallel (Siamese network) units capture distinct features corresponding to the ratio of white matters and gray matters. The fusion in the middle outperformed competitors’ fusions (Fig. 2 and Supplementary Table 4). The second row (Fig. 5) highlights asymmetric and symmetric features. After merging ($\oplus$), the model retains the contrastive and symmetrical information necessary for the CP identification. Notable SMSeq-DL can be employed for MS-MRI and SS-MRI with multiple copies.

#### CP vulnerable slices in MRI scans

**Fig. 6:**
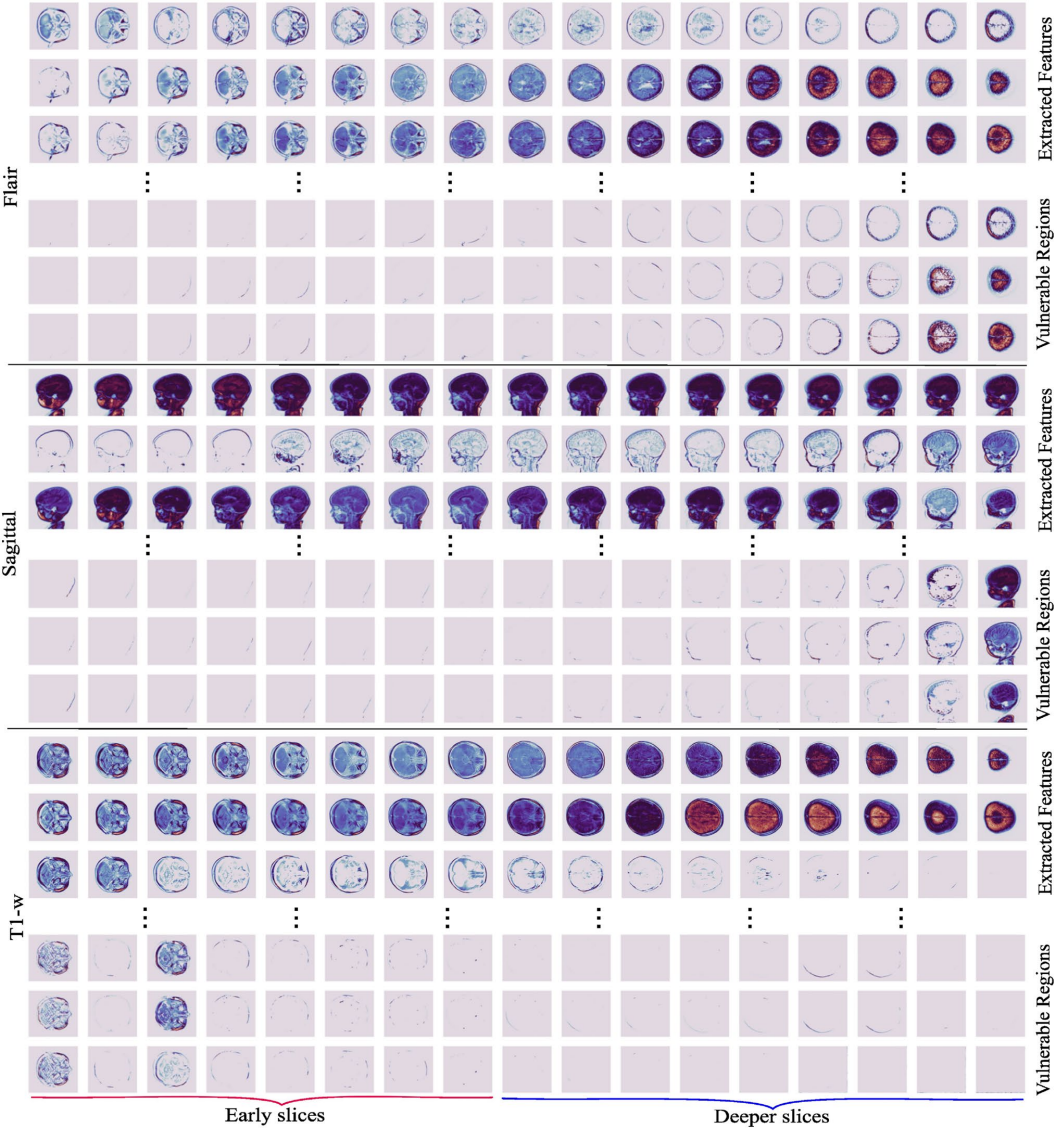
The three MRI scans, including Flair, Sag, and T1-w, have been depicted with unique learning trends. In each MRI scan, the first row represents generalized captured features by the DL model, while the second row shows the focused or salient feature maps

In clinical practices, it is inevitable to utilize attention-like mechanisms to exploit the black box-like notorious attribute of DL architectures. Therefore, the abstract visualizations have been shown for Flair, Sag, and T1-w scans (Fig. 6). Each scan has two types of visuals corresponding to the prior (first row) and post (second row)-employing attention. In the Flair scan case, the model focuses on deeper slices, which are shown in the second row, for CP identification. A similar trend can be observed for Sag, where the last few slices receive more attention. Contrarily, in the case of the T1-w scan, the model learns features from the early slices and discards unnecessary information (see details in Supplementary Figures 11, 12, and 13). For T2-w contrast, the model extracts features randomly (Supplementary Figure  10). The coupling of T2-w$\oplus$Flair has been used for elaborating trends between controls and CP.

**Fig. 7:**
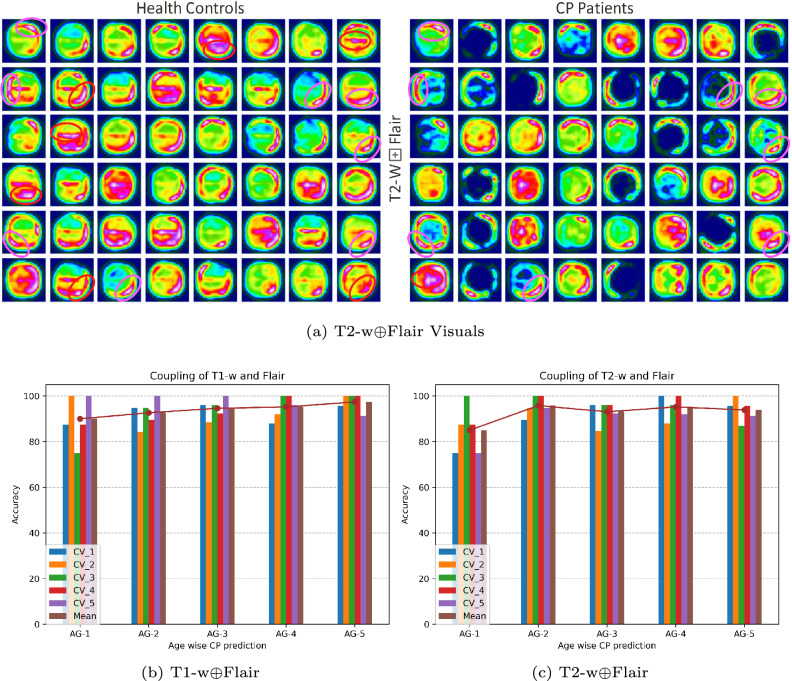
First row: Visualization of trends for health controls and CP subject using T2-w$\oplus$Flair coupling. Second row: Age-wise CP prediction scores in coupling T1-w$\oplus$Flair and T2-w$\oplus$Flair. Each group ran for fivefold cross-validations (CV_1, CV_2, CV_3, CV_4, and CV_5)

Seventeen slices are grouped into four categories (Supplementary Figure 3 and Tables 1, 2). For instance, the visual depictions show that CP is deeply associated with Sag in the deeper slices (Fig. 6-second row). The brain tissues covered in those slices include the temporal, lateral, parietal, occipital, angular, insula, cerebellum, and hippocampus (Supplementary Figure 13). Similarly, for the depiction of T1-w, we deduce that the most vulnerable regions include the Insula, Cerebellum, Superior Motor Area on the left side, and Hippocampus (Fig. 7).

#### Trends in health controls and CP

Besides depicting lesion-vulnerable regions in brain MRI in each scan, it is also inevitable to show generalized features captured by the DL against health controls and CP patients. Therefore, we employed the coupling of T2-w$\oplus$Flair to highlight the RoIs corresponding to lesions in the case of CP and generic features fall for health controls distinct from CP (Fig. 7a). The red spheroid-covered regions represent different features, whereas the pink spheroid denotes symmetric features. The asymmetric features between the two groups help the DL model identify CP and health controls where a particular MRI scan distinction can be made using symmetric features.

### CP identification from infancy to adolescence

CP prediction from infancy to adolescence is critical in radiomics for CP examination. Therefore, this section aims to elaborate on the CP prediction from an early age (a few months) to the age of 17 years. The testing samples are grouped into five age-wise (AG) groups (Fig. 7b, c) (see details in Supplementary). The first five vertical bars show the fivefold cross-validations, whereas the last bar shows the average of each age-wise group for the cross-validations. For result evaluation, only T1-w$\oplus$Flair (Fig. 7b) and T2-w$\oplus$Flair (Fig. 7c) are considered because of their superior performances. In the evaluation of T1-w$\oplus$Flair (Fig. 7b), the overall CP prediction score is lower, whereas the cross-validation statistics show irregular accuracy measures. It extrapolates rapid brain development at an early age compared to adolescence. This trend continues to the later age groups (AG-5), where more consistent CP trends are observed with promising prediction accuracy. Furthermore, the findings of T2-w$\oplus$Flair (Fig. 7c) in the early age (AG-1) show a similar trend to that of T1-w$\oplus$Flair (Fig. 7b). However, there is a high prediction score for AG-2, which continues smoothly to AG-5. From the models’ training findings, the accumulative CP identification score was distinct from the early age (AG-1) and later age (AG-5).

## Conclusion

The two novels introduced models, SSeq-DL and MS-DL, trained on SS-MRI and MS-MRI and robustly classify CP and controls. The accuracy of CP identification using SSeq-DL (SS-MRI) reached 90%, which further improved to around 94% using SMSeq-DL (MS-MRI). Multiple scans retain complementary information and vulnerable regions to increase CP prediction. Among SS-MRI, the T2-w results in a higher prediction score compared to competitor scans, whereas the Sag scan is reported poorly. Similarly, from the possible couplings, T1-w$\oplus$Flair and T2-w$\oplus$Flair were reported with superior results; however, Flair in isolated form is reported with less significant results. Poor performance was also observed when coupling with the Sag scan. T1-w$\oplus$Flair mainly focuses on the early slices along the depth to predict CP. All the slices of T2-w$\oplus$Flair receive more attention. The DL models trained on the joint adventure of Sag mainly focused on the last deeper slices and hence produced poor results. From the visualized results, the vulnerable MRI slices associated with CP spread to the brain regions, including the temporal, rhizomatic, parietal, occipital, angular, insula, cerebellum, hippocampus, and superior motor area on the left side of the brain. CP and controls reported symmetric and asymmetric trends. In age-wise, inconsistent trends were reported at an early age, deducing the rapid brain development from infancy to adolescence. The proposed research is limited to CP, which can be extended to other neurological disorders. In addition, the study is limited to a solitary research hospital, but it has the potential to be expanded and integrated with additional research hospitals.

## Supplementary Information

Below is the link to the electronic supplementary material.

Supplementary file 1 (pdf 48098 KB)

## References

[1] Accardo J, Kammann H, Hoon AH Jr (2004) Neuroimaging in cerebral palsy. J Pediatr 145:S19–S27 15292883 10.1016/j.jpeds.2004.05.018

[2] Akçakaya M, Moeller S, Weingärtner S, Uğurbil K (2019) Scan-specific robust artificial-neural-networks for k-space interpolation (RAKI) reconstruction: database-free deep learning for fast imaging. Magn Reson Med 81:439–453 30277269 10.1002/mrm.27420 PMC6258345

[3] Andermatt S, Pezold S, Cattin PC (2018) Automated segmentation of multiple sclerosis lesions using multi-dimensional gated recurrent units. In: Brainlesion: glioma, multiple sclerosis, stroke and traumatic brain injuries: third international workshop, BrainLes 2017, Held in Conjunction with MICCAI 2017, Quebec City, QC, Canada, September 14, 2017, Revised Selected Papers 3, Springer. pp. 31–42

[4] Bahado-Singh RO, Vishweswaraiah S, Aydas B, Mishra NK, Guda C, Radhakrishna U (2019) Deep learning/artificial intelligence and blood-based DNA epigenomic prediction of cerebral palsy. Int J Mol Sci 20:2075 31035542 10.3390/ijms20092075 PMC6539236

[5] Bertoncelli CM, Altamura P, Vieira ER, Bertoncelli D, Solla F (2019) Using artificial intelligence to identify factors associated with autism spectrum disorder in adolescents with cerebral palsy. Neuropediatrics 50:178–187 31018221 10.1055/s-0039-1685525

[6] Bertoncelli CM, Altamura P, Vieira ER, Iyengar SS, Solla F, Bertoncelli D (2020) Predictmed: a logistic regression-based model to predict health conditions in cerebral palsy. Health Inf J 26:2105–2118 10.1177/1460458219898568 31957544

[7] Groos D, Adde L, Aubert S, Boswell L, De Regnier RA, Fjørtoft T, Gaebler-Spira D, Haukeland A, Loennecken M, Msall M et al (2022) Development and validation of a deep learning method to predict cerebral palsy from spontaneous movements in infants at high risk. JAMA Netw Open 5:e2221325–e2221325 35816301 10.1001/jamanetworkopen.2022.21325 PMC9274325

[8] Gupta A, Al-Dasuqi K, Xia F, Askin G, Zhao Y, Delgado D, Wang Y (2017) The use of noncontrast quantitative MRI to detect gadolinium-enhancing multiple sclerosis brain lesions: a systematic review and meta-analysis. Am J Neuroradiol 38:1317–1322 28522663 10.3174/ajnr.A5209 PMC5509500

[9] Herskind A, Greisen G, Nielsen JB (2015) Early identification and intervention in cerebral palsy. Dev Med Child Neurol 57:29–36 25041565 10.1111/dmcn.12531

[10] Illavarason P, Arokia Renjit J, Mohan Kumar P (2019) Medical diagnosis of cerebral palsy rehabilitation using eye images in machine learning techniques. J Med Syst 43:1–24 10.1007/s10916-019-1410-6 31289923

[11] Krägeloh-Mann I (2008) Understanding causation of cerebral palsy by using magnetic resonance imaging. Paediat Child Health 18:399–404

[12] Krigger KW (2006) Cerebral palsy: an overview. Am Fam Phys 73:91–100 16417071

[13] La Rosa F, Fartaria MJ, Kober T, Richiardi J, Granziera C, Thiran JP, Cuadra MB (2019) Shallow vs deep learning architectures for white matter lesion segmentation in the early stages of multiple sclerosis. In: Brainlesion: glioma, multiple sclerosis, stroke and traumatic brain injuries: 4th international workshop, BrainLes 2018, Held in Conjunction with MICCAI 2018, Granada, Spain, September 16, 2018, Revised Selected Papers, Part I 4, Springer. pp. 142–151

[14] Lee-Park JJ, Deshpande H, Lisinski J, LaConte SM, Ramey SL, DeLuca SC (2018)Neuroimaging strategies addressing challenges in using FMRI for the children with cerebral palsy

[15] MacLennan AH, Thompson SC, Gecz J (2015) Cerebral palsy: causes, pathways, and the role of genetic variants. Am J Obstet Gynecol 213:779–788 26003063 10.1016/j.ajog.2015.05.034

[16] McIntyre S, Morgan C, Walker K, Novak I (2011) Cerebral palsy? Don’t delay. Dev Disabil Res Rev 17:114–129 23362031 10.1002/ddrr.1106

[17] Miller F, Bachrach SJ (2017) Cerebral palsy: a complete guide for caregiving. JHU Press, Baltimore

[18] Palraj P, Siddan G (2021) Deep learning algorithm for classification of cerebral palsy from functional magnetic resonance imaging (fmri). Int J Adv Comput Sci Appl. 10.14569/IJACSA.2021.0120383

[19] Reid SM, Dagia CD, Ditchfield MR, Carlin JB, Meehan EM, Reddihough DS (2014) An Australian population study of factors associated with MRI patterns in cerebral palsy. Dev Med Child Neurol 56:178–184 24428267 10.1111/dmcn.12331

[20] Saha S, Pagnozzi A, Bourgeat P, George JM, Bradford D, Colditz PB, Boyd RN, Rose SE, Fripp J, Pannek K (2020) Predicting motor outcome in preterm infants from very early brain diffusion MRI using a deep learning convolutional neural network (CNN) model. Neuroimage 215:116807 32278897 10.1016/j.neuroimage.2020.116807

[21] Sakkos D, Mccay KD, Marcroft C, Embleton ND, Chattopadhyay S, Ho ES (2021) Identification of abnormal movements in infants: a deep neural network for body part-based prediction of cerebral palsy. IEEE Access 9:94281–94292

[22] Tortora D, Panara V, Mattei P, Tartaro A, Salomone R, Domizio S, Cotroneo A, Caulo M (2015) Comparing 3t t1-weighted sequences in identifying hyperintense punctate lesions in preterm neonates. Am J Neuroradiol 36:581–586 25376807 10.3174/ajnr.A4144 PMC8013055

[23] Towsley K, Shevell MI, Dagenais L, Consortium R et al (2011) Population-based study of neuroimaging findings in children with cerebral palsy. Eur J Paediat Neurol 15:29–35 10.1016/j.ejpn.2010.07.005 20869285

[24] Wang J, Shen X, Hu X, Yang H, Yin H, Zhu X, Gao H, Wu Y, Meng F (2021) Early detection relationship of cerebral palsy markers using brain structure and general movements in infants born< 32 weeks gestational age. Early Human Dev 163:105452 10.1016/j.earlhumdev.2021.105452 34543944

[25] Yang R, Zuo H, Han S, Zhang X, Zhang Q (2021) Computer-aided diagnosis of children with cerebral palsy under deep learning convolutional neural network image segmentation model combined with three-dimensional cranial magnetic resonance imaging. J Healthc Eng 2021:1822776 34804446 10.1155/2021/1822776 PMC8598324

[26] Yoshida S, Hayakawa K, Oishi K, Mori S, Kanda T, Yamori Y, Yoshida N, Hirota H, Iwami M, Okano S et al (2011) Athetotic and spastic cerebral palsy: anatomic characterization based on diffusion-tensor imaging. Radiology 260:511–520 21555354 10.1148/radiol.11101783

[27] Cy Zhang, Bf Yan, Mutalifu N, Yw Fu, Shao J, Jj Wu, Guan Q, Biedelehan Sh, Lx Tong, Xp Luan (2022) Predicting the brain age of children with cerebral palsy using a two-dimensional convolutional neural networks prediction model without gray and white matter segmentation. Front Neurol 13:1040087 36504669 10.3389/fneur.2022.1040087 PMC9730825

[28] Zhang J (2017) Multivariate analysis and machine learning in cerebral palsy research. Front Neurol 8:715 29312134 10.3389/fneur.2017.00715 PMC5742591

[29] Zhang Y, Ma Y (2019) Application of supervised machine learning algorithms in the classification of sagittal gait patterns of cerebral palsy children with spastic diplegia. Comput Biol Med 106:33–39 30665140 10.1016/j.compbiomed.2019.01.009

[30] Zheng H, Qu X, Bai Z, Liu Y, Guo D, Dong J, Peng X, Chen Z (2017) Multi-contrast brain magnetic resonance image super-resolution using the local weight similarity. BMC Med Imaging 17:1–13 28095792 10.1186/s12880-016-0176-2 PMC5240324

[31] Himmelmann K, Horber V, Sellier E, De la Cruz J, Papavasiliou A, Krägeloh-Mann I et al (2021) Neuroimaging patterns and function in cerebral palsy-application of an MRI classification. Front Neurol 11:617740 33613420 10.3389/fneur.2020.617740 PMC7887285

[32] Hinchberger V, Kang SH, Kline J, Stanley CJ, Bulea TC, Damiano DL (2023) Investigation of brain mechanisms underlying upper limb function in bilateral cerebral palsy using EEG. Clin Neurophysiol 151:116–127 37245498 10.1016/j.clinph.2023.04.006 PMC10330582

[33] Zhu M, Men Q, Ho ES, Leung H, Shum HP (2021) Interpreting deep learning based cerebral palsy prediction with channel attention. In: 2021 IEEE EMBS international conference on biomedical and health informatics (BHI), IEEE. pp. 1–4

[34] Szkoda L, Szopa A, Kwiecień-Czerwieniec I, Siwiec A, Domagalska-Szopa M (2023) Body composition in outpatient children with cerebral palsy: a case-control study. Int J General Med 16:281–291 10.2147/IJGM.S393484 PMC9883993 36718145

[35] Leonard JM, Cozens AL, Reid SM, Fahey MC, Ditchfield MR, Reddihough DS (2011) Should children with cerebral palsy and normal imaging undergo testing for inherited metabolic disorders? Dev Med Child Neurol 53:226–232 21291466 10.1111/j.1469-8749.2010.03810.x

[36] Benini R, Dagenais L, Shevell MI, Registre de la Paralysie Cérébrale au Québec (Quebec Cerebral Palsy Registry) Consortium (2013) Normal imaging in patients with cerebral palsy: What does it tell us? J Pediatr 162:369–374 22944004 10.1016/j.jpeds.2012.07.044

[37] Krägeloh-Mann I, Horber V (2007) The role of magnetic resonance imaging in elucidating the pathogenesis of cerebral palsy: a systematic review. Dev Med Child Neurol 49:144–151 17254004 10.1111/j.1469-8749.2007.00144.x

[38] Mohan PP, Ramkumar G (2024) Experimental evaluation of brain cerebral palsy disease prediction using artificial intelligence assisted learning methodology. In: 2024 ninth international conference on science technology engineering and mathematics (ICONSTEM), IEEE. pp. 1–7

[39] Lima AA, Mridha MF, Das SC, Kabir MM, Islam MR, Watanobe Y (2022) A comprehensive survey on the detection, classification, and challenges of neurological disorders. Biology 11:469 35336842 10.3390/biology11030469 PMC8945195

[40] Malagi AV, Netaji A, Kumar V, Baidya Kayal E, Khare K, Das CJ, Calamante F, Mehndiratta A (2022) Ivim-dki for differentiation between prostate cancer and benign prostatic hyperplasia: comparison of 1.5 t vs. 3 t MRI. Magn Reson Mater Phys, Biol Med 35:609–620 10.1007/s10334-021-00932-1 34052899

[41] Tajima T, Akai H, Yasaka K, Kunimatsu A, Yoshioka N, Akahane M, Ohtomo K, Abe O, Kiryu S (2023) Comparison of 1.5 t and 3 t magnetic resonance angiography for detecting cerebral aneurysms using deep learning-based computer-assisted detection software. Neuroradiology 65:1473–1482 37646791 10.1007/s00234-023-03216-8

[42] Kushol R, Parnianpour P, Wilman AH, Kalra S, Yang YH (2023) Effects of MRI scanner manufacturers in classification tasks with deep learning models. Sci Rep 13:16791 37798392 10.1038/s41598-023-43715-5 PMC10556074

[43] Li X, Morgan PS, Ashburner J, Smith J, Rorden C (2016) The first step for neuroimaging data analysis: DICOM to NIfTI conversion. J Neurosci Methods 264:47–56 26945974 10.1016/j.jneumeth.2016.03.001

