# Supplementary material for: Trends in brain MRI and CP association using deep learning
Source: Radiol Med. 2024 Oct 10;129(11):1667–81. doi: 10.1007/s11547-024-01893-w (PMC11554846; doi:10.1007/s11547-024-01893-w)
Supplement: Supplementary file 1 — (pdf 48098 KB) [file 11547_2024_1893_MOESM1_ESM.pdf]

Trends in Brain MRI and CP Association Using Deep Learning

Abstract

Cerebral palsy (CP) is a neurological disorder that dissipates body posture and impairs motor functions. It may lead to an intellectual disability and affect the quality of life. Early intervention is critical and challenging due to the uncooperative body movements of children, potential infant recovery, a lack of a single vision modality, and no specific sequence or slice-range selection and association. Early and timely CP identification and vulnerable brain MRI scan associations facilitate medications, supportive care, physical therapy, rehabilitation, and surgical interventions to alleviate symptoms and improve motor functions. The literature studies are limited in selecting appropriate sequence and scan and utilizing contrastive coupling in CP investigation. After numerous experiments, we introduce deep learning models, namely SSeq-DL and SMSeq-DL, correspondingly trained on single sequence and multiple brain MRIs. The introduced models are tailored with specialized attention mechanisms to learn susceptible brain trends associated with CP along the MRI slices, specialized parallel computing, and fusions at distinct network layer positions to significantly identify CP. The study successfully experimented with the appropriateness of single and coupled MRI scans, highlighting sensitive slices along the depth, model robustness, fusion of contrastive details at distinct levels, and capturing vulnerabilities. The findings of the SSeq-DL and SMS-DL models report lesion-vulnerable regions and covered slices trending in age range to assist radiologists in early rehabilitation.

Keywords: Cerebral Palsy, Early Intervention, MRI Coupling, Vulnerabilities, Deep Learning

Supplementary Materials

0.1 MRI slices distribution

0.1.1 MRI view position

The dataset used in this investigation included T1-w, T1-sag, T2-w, and Flair contrasts (sequences) images obtained from axial or sagittal perspectives. The brain MRI's axial view may reveal distinct brain tissues based on the depth of the slice. We may examine labeled regions in Figure M-3(a)from the central section to provide a visual representation. The MIRcron software tool demonstrated a distinct differentiation (slice) among the included regions. Furthermore, we provide the sagittal perspective of the brain MRI, as shown in Figure M-3(b).The sagittal slice depicts several brain regions, with the number of regions shown (up to 8 for illustrative purposes) dependent on the slice's depth. The axial view is used for the T1-w, T2-w, and Flair scans. The sagittal view of the brain is used for T1-sag contrast, which reveals distinct depths and generates regions of interest. The brain structure's covered regions in a slice are crucial for machine learning to capture disorder-associated areas. These viewings aim to expedite the assessment of sensitive brain areas from different points of view. The attention mechanism of the suggested model is primarily focused on these sensitive areas, which are visualized in the later section.

0.1.2 Axial and sagittal views based slices distribution

Before preprocessing, variations in the dimensions of the 3D MRI were obtained from the subjects and controls. Therefore, aligning the dimensions into the same dimension is inevitable. All the MRIs are resized to the same depth as the slice number, i.e., 17. We divide the slice into four groups, both for axial view (Table 1) and sagittal view (Table 2) in order to easily understand the sensitive regions representing lesions. The first to the fourth group correspondingly contains 1-4, 5-8, 9-13, and 13-17 slices. This distribution applies to both axial view (Figure M-3(a),Table 1) and sagittal view (Figure M-3(b).

Table 1: The MRI slices obtained from axial view are divided into 4 groups. Each group has shown its key regions covered along the slices.

Scans 1-4	Scans 5-8	Scans 9-13	Scans 14-17
Cerebelum, 1-10, L-R	Cerebelum, 1-10, L-R	—	—
Spinal, Trapezius	—	—	—
Rectus capitis post	Rectus capitis, L-R	Rectus, L-R	—
Foramen magnum	—	—	—
Vertebral artery	—	—	—
Occipital condyle-bone	Occipital, Inf, L-R	Occipital,Mid, Inf, Sup, L-R	—
Medial pterygoid, Stylopharyngeus	—	—	—
Styloglossus, Longus capitis	—	—	—
Uvula, Atlas, Masseter	—	—	—
Parotid gland, Orbicularis aris	—	—	—
Sternocleidomastoid	—	—	—
Foramen magnum	—	—	—
Temporal, L-R	Temporal, L-R	Temporal, L-R	—
Fusiform, L-R	Fusiform, L-R	Fusiform, L-R	—
Fourth ventricle	Third Fourth ventricle, L-R	Third Lateral ventricle, L-R	Lateral ventricle L-R
—	Pone, Amygdala, L-R	—	—
—	Hippocampus, L-R	—	—
—	Calcarine, L-R	Calcarine, L-R	—
—	Frontal, Ope, Inf, Sup, Medial, Tri, L-R	Frontal, Ope, Inf, Sup, Medial, Tri, L-R	—
—	Insula, L-R	Insula, L-R	—
—	—	Cingulum, Ant, Post, Mid, L-R	—
—	—	Supp Motor, Area, L-R	Supp Motor, Area, L-R
—	—	Precuneus, L-R	Precuneus, L-R
—	—	Parietal, Sup, L-R	Parietal, Sup, L-R

Inferior (Inf), Middle (Mid), Anterior (Ant), Posterior (Post), Left (L), Right (R), Orbit (Orb), Operculum (Oper)

Table 2: The division of Sag viewed MRI's slices into 4 groups. Each group outlines the regions of interest that are used in the visual evaluation section.

Scans 1-4	Scans 5-8	Scans 9-13	Scans 14-17
Sup-Marginal, L	—	—	Sup-Marginal, R
Postcentral, L	—	—	Postcentral, R
Rolandic, Oper, L	—	Rolandic, Oper, L	Rolandic, Oper, R
Temporal, Sup, Mid, Inf, L	—	—	Temporal, Inf, Pole, Mid, Sup, R
Frontal, Mid, Inf, Orb	Frontal, Mid, Inf, Orb	Frontal, Mid, Inf, Orb	Frontal, Mid, Inf, Orb
Parietal, Inf, L	Parietal, Inf, L	Parietal, Sup, R	Parietal, Sup, R
Occipital, Mid, Sup, L	Occipital, Sup, L	Occipital, Sup, R	Occipital, Sup, R
Angular, L	—	Angular, L	Angular, R
Precentral, L	—	Precentral, L	Precentral, R
Fusiform, L	Fusiform, L	—	Fusiform, R
Heschi, L	—	—	Heschi, R
Insula, L	—	—	Insula, R
Cerebelum, L	Cerebelum, L	Cerebelum, R	Cerebelum, R
Supp, Motor, Area, L	Supp, Motor, Area, L-R	Supp, Motor, Area, L-R	—
—	Cingulum, L	Cingulum, L	—
—	Thalamus, L	Thalamus, L-R	—
—	—	Vemis 3-8, Corpus callosum	—
—	—	Pons 3-4,Pituitary gland	—
—	Pallidum, L	Pallidum, R	Pallidum, R
—	Amygdala, L	Amygdala, R	—
Hippocampus, L	Hippocampus, L	Hippocampus, R	Hippocampus, R

Inferior (Inf), Middle (Mid), Anterior (Ant), Posterior (Post), Left (L), Right (R),Orbit (Orb), Operculum (Oper)

0.2 Single Scan MRI Based Modeling

In this study, we conducted several experiments to evaluate the appropriateness of each MRI scan for CP prediction. After extensive model training, we chose seven DL models based on their number of parameters, efficiency, and performance. All the network model parameters are shown in Table 3.

Table 3: Single MRI scan based deep learning models' parameters.

Model	Conv.Layers	Augmentation	Attention
S-Model-1	5-Conv.Layer+5-MaxPool+3-F.C	✗	✗
S-Model-2	5-Conv.Layer+5-MaxPool+3-F.C	✓	✗
S-Model-3	5-Conv.Layer+5-MaxPool+3-F.C+CA	✓	✓
S-Model-4	5-Conv.Layer+5-MaxPool+3-F.C+SA	✓	✓
S-Model-5	5-Conv.Layer+5-MaxPool+3-F.C+CA+SA+Seq	✓	✓
S-Model-6	5-Conv.Layer+5-MaxPool+3-F.C+CA+SA+Paral	✓	✓
S-Model-7	5-Conv.Layer+5-MaxPool+3-F.C+CA+SA+Paral+Deeper	✓	✓

Convolution layer (Conv.Layer), Fully connected (F.C), Channel attention (CA), Spatial attention (SA), Sequential (Seq), Parallel (Paral)

The training flow of each DL model over SS-MRI is shown in Figure 1. The first model (S-Model-1) trains with simple 3D convolution operations, downsampling, non-linear activation functions, and fully connected layers. However, no augmentation or attention is applied to the network structure (Figure 1(a)). In the same way, to make S-Model-1 better at learning, the second model (S-Model-2) uses augmentation (Figure 1(b)). The experts and clinicians have verified to avoid the augmentation-based downgrading effects vulnerable to brain MRI. In the same way, S-Model-3 uses a focus mechanism (channel attention-AM-C) to draw attention to the critical part along the channel-wise axis (Figure 1(c)). Similarly, S-Model-4 utilizes spatial attention (AM-S) to capture features along spatial dimensions (Figure 1(c)). The S-Model-5 applies both AM-C and AM-S in sequential order (Figure 1(d)), while the S-Model-6 incorporates AM-C and AM-S in parallel, which is later on chosen as SSeq-DL based on its performance (Figure 1(e)). Finally, S-Model-7 applies a deeper network to increase the models' learning capacity in either case. We train the seven proposed models on each MRI sequence, such as T1-w, T1-sag, T2-w, and Flair (Table 2).

Among the list of SS-MRI-based DL models, the robust architecture as SSeq-DL is shown in main Figure 1. The architecture of SSeq-DL is equipped with the appropriate placement of both spatial (SA) and channel (CA) attention (SCA). However, CA, followed by SA, was found more suitable based on the promising CP identification results. The placement of the early layers of SCA twice in the architecture shows appropriateness compared to the placement of the deeper layer. SSeq-DL receives only a single scan (T1-w, T1-sag, T2-w, and Flair) and predicts CP from it. The input tensor is passed into the convolution layer, which has 24 feature maps and a kernel size of 5, to process the MRI scan. The output is directed to the spatial and channel-wise attention (SCA-1), where it is combined with the residual connection via element-wise merging. The resulting output is subsequently delivered to the convolution relu and max pool (CRM-1) block. The output of CRM-1 is combined with SCA-2 via element-wise merging. The characteristics of SCA are shown in the channel block (CA), followed by the specialized spatial attention (SP). A stacked of CRM (CRM-2,3,4) is used, followed by fully connected layers that include drop-out layers, in order to make predictions for CP.

0.3 Learning from Multiple Brain MRI Scans

0.3.1 Shifting From Single to Multiple Scans Based Learning

In the preceding part, we endeavored to determine the most effective deep learning model for single scan MRI in the examination of neurological disorders such as CP. However, this section delineates the network architecture that addresses parallel computing in order to enhance prediction accuracy. In the parallel computing or partial siamese learning unit, the pipeline receives multiple MRI scans

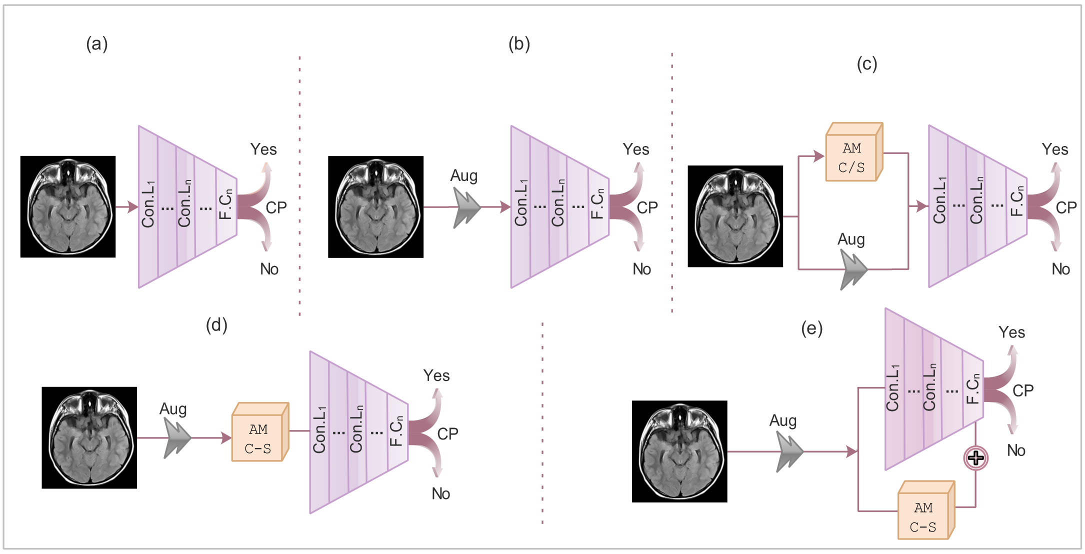

AM C/S: Attention Mechanism (Channel or Spatial), **AM C-S**: Attention Mechanism (Channel and Spatial), **Aug**: Augmentation, **Con.L**: Convolution layer, **F.C**: Fully connected, **CP**: Cerebral Palsy

Fig. 1: A total of seven DL models have been visualized in five small figures. The models are trained on different MRI scans (T1-w, T1-sagittal, T2-w, and Flair) to determine the most appropriate DL model for C.P. (a) The model is composed of convolutional layers (Con.L.), downsampling, and a non-linear activation function, and ends with fully connected layers (F.C.). (b) Models apply augmentation in the learning process. (c) The model represents two DL concepts with spatial (AM-S) or channel (AM-C) attention mechanisms. (d) The model utilizes a parallel channel-spatial mechanism in a sequential order. (e) The model employs channel-spatial attention (AM C-S). The parallel AM can be applied at distinct depth levels.

(multi-sequence) for sharing and learning to share complementary information (Figure 2). For a single MRI, a number of scans may be inputted into the parallel processing unit for multiple copies. The presence or absence of certain characteristics in the Siamese network would greatly enhance the accuracy of CP prediction. Nevertheless, we use a partial siamese network to generate a more versatile model and prevent susceptibility in handling any version ofinputMRI. The partial siamese fuses the parallel units at mid-level to allow the network to comprehend prior and post-fusions. The model retains the weights of each input scan to the mid-level, then shares the common weights, utilizes discrepant features to enhance robustness in class imbalance, and tackles fewer samples per class. Therefore, the deeper study aims to shift from **s**ingle to **m**ultiple **s**equence (contrast) based **DL** modeling (SMSeq-DL) (Figure 2) to benchmark against the competitors including multiple levels of fusions (early, mid, and late) modeling (Figure 1)(a-e), SSeq-DL (Figure 1)(e), and state-of-the-art (SOTA). Therefore, we run numerous experiments underlying the possible sequence (Figure 1). Before sharing the weights at a particular level, capturing the most vulnerable brain regions is necessary to optimally identify neurological disorders, specifically CP. Therefore, the spatial and channel attentions are employed with different variants to outline the exciting features associated with CP in brain MRI (Figure 1). Based on the significant results, we incorporate channel and spatial attention in a parallel or sequential fashion under the umbrella of siamese structure. Similarly, incorporating attention mechanisms before and after fusions outperformed counterpart variants for CP estimations. Likewise, we ran experiments to determine the best modelling based on spatial only, channel only, spatial and channel in parallel, and spatial and channel in sequential (Figure 2).

The MS-MRI can visualize white matter and grey matter, and their complementary effects reflecting CP-related variations can be captured through a well-designed DL architecture. The

SMSeq-DL architecture comprises parallel computing, partial siamese, fusion modeling, and attention mechanisms. The network architecture copes with noise and artifact challenges by training on cleaned and augmented datasets. Similarly, using SMSeq-DL, clinicians and domain experts can predict and diagnose CP early and accurately, which is often vulnerable to infants' age and tiny lesions. This study establishes a benchmark study based on theoretical grounds and clinically beneficial in the domain.

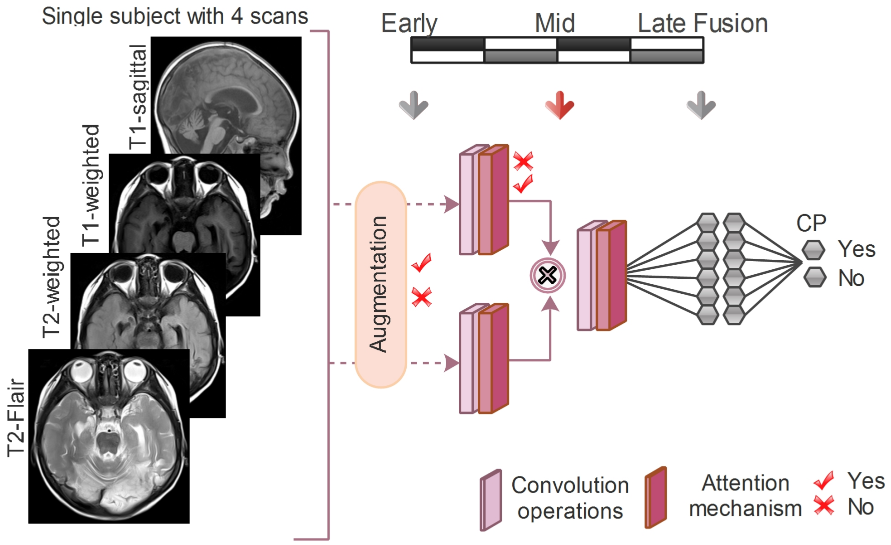

Fig. 2: Our proposed DL model (SMSeq-DL) receives MS-MRI. The SMSeq-DL employs appropriate augmentation before training and evaluation. The augmented MS-MRI forwards to a parallel computing environment (siamese network), followed by fusions. Based on the results' significance, the attention mechanism is employed after normal convolution operations at an appropriate place. Finally, the fully connected layers in SMC pass the learned weights to predict CP in MRI images.

0.3.2 Complementary Features Utilization from Multiple MRI Scans

The multi-sequence MRI (MS-MRI) in raw form can be acquired by following the physics rules [12] as by recalling main Equation 3. By setting different values of TR and TE, MS-MRI images will be acquired, sharing anatomical structures and proton density of the subjects' MRI but with varying contrasts in regions. The acquired multi-scan MRI MR_m for single subject forwards for learning CP-related patterns together with the contrastive information formulated as follows:

$$SMSeq - DL = \sum_{i}^{j} MC_i\{MR_m : \;\; \Rightarrow \; \Upsilon \rightarrow (Ni_p, \oplus || \otimes, \;\; Ni_{p+1}) \;\; \prec \ell_d(Ni_p \odot Ni_{p+1}), AM, \omega, \beta\}, \tag{1}$$

The multi-contrastive learning MC applies to a batch of MS-MRI in the range of $i \rightarrow j$. The preprocessing Υ versions of MR_m pass to the partially parallel units, such as Ni_p and Ni_{p+1}, and merge either with elementwise sum $\oplus$ or multiplication $\otimes$. The merging operation $\prec$ carries at different depth levels ℓ_d and proceeds to a joint $(Ni_p \odot Ni_{p+1})$ feature representation. Similarly, each network may apply attention mechanisms (AM) at different positions depending on the network's learned weights ω and biases β.

The network parameters of SMSeq-DL (Figure 2) are shown in Table 4. The models' structure is composed of a convolution layer (Conv. Layer), channel attention (CA), spatial attention (SA), sequential (Seq), parallel (Paral), max-pool (MP), and fully connected (FC). In contrast, each fully connected layer is followed by a drop-out layer to avoid overfitting.

Table 4: Multi-contrast MRI scan based deep learning models' parameters.

Model	Conv.Layers		
M-Model-1	C.L-24×7, M.P-8×4→C.L-48×5, M.P-8×4<br>C.L-24×7, M.P-8×4→C.L-48×5, M.P-8×4	⊗	C.L-96×5, M.P-4×2→ C.L-192×3, M.P-4×2→F.C512-8, 384-7, 256-6, 2
M-Model-2	C.L-24×7, M.P-8×4→C.L-48×5, M.P-8×4<br>C.L-24×7, M.P-8×4→C.L-48×5, M.P-8×4	⊕	C.L-96×5, M.P-4×2→ C.L-192×3, M.P-4×2→F.C512-8, 384-7, 256-6, 2
M-Model-3	C.L-24×7-SA, M.P-8×4→C.L-48×5, M.P-8×4<br>C.L-24×7-SA, M.P-8×4→C.L-48×5, M.P-8×4	⊕	C.L-96×5, M.P-4×2→ C.L-192×3, M.P-4×2→F.C512-8, 384-7, 256-6, 2
M-Model-4	C.L-24×7-SA, M.P-8×4→C.L-48×5, M.P-6×4<br>C.L-24×7-SA, M.P-8×4→C.L-48×5, M.P-6×4	⊕	C.L-96×5, M.P-4×2→ C.L-192×3, M.P-4×2→F.C384-7, 256-6, 2
M-Model-5	C.L-24×7, M.P-8×4→C.L-48×5-SA, M.P-8×4<br>C.L-24×7, M.P-8×4→C.L-48×5-SA, M.P-8×4	⊕	C.L-96×5, M.P-4×2→ C.L-192×3, M.P-4×2→F.C512-8, 384-7, 256-6, 2
M-Model-6	C.L-24×7-SA, M.P-8×4<br>C.L-24×7-SA, M.P-8×4	⊕	C.L-48×5, M.P-8×4, C.L-96×5, M.P-4×2→ C.L-192×3, M.P-4×2→F.C512-8, 384-7, 256-6, 2

Convolution layer (Conv.Layer), Fully connected (F.C), Channel attention (CA), Spatial attention (SA), Sequential (Seq), Parallel (Paral), Max-pool (MP).

0.4 Dataset collection

The previous studies have collected a small number of CP patients (70 children) for training a DL model [51]. However, enough CP patients are needed to train a reliable DL model. To our knowledge, this study collects the first largest CP dataset [60]. There are 716 subjects; 327 are patients, and 389 are controls. The control participants' MRIs were obtained using Skyra, GE, and Phillips machines, with ratios of 123, 138, and 128, respectively. However, most patients' MRIs were obtained using GE (282), while a minority of patients' MRIs were recorded using Skyra (44). The table below (Table 5) provides the statistical distribution of the acquired MRI data. MRI images captured at different field strengths vary in signal-to-noise ratio (SNR), contrast, resolution, and susceptibility to artefacts. The magnetic field strength with variations influences CNN-based DL models and, therefore, can focus on learning small anatomical structures in the brain [60–62].

Table 5: Skyra, GE, and Phillips MRI scanners based dataset collection and comparison.

MRI Machine	HC	Patient	PSNR	Contrast between tissues	Information potential	Anatomical details
Skyra (3T)	123	44	High	High	High	High
GE (1.5)	138	282	Low	Low	Low	Low
Philips (3T)	128	1	High	High	High	High
Total	389	327				

The diverse training datasets, including images from 1.5T and 3T MRI scanners, can help develop more robust training and reliable models [63]. Table 6 presents factors that affect DL training, efficiency, and robustness between single-scanner and multi-scanner datasets. Higher field strengths, like 3T, generally provide images with higher PSNR and more excellent contrast between different tissue types, potentially offering more detailed information for deep learning models to learn. Therefore, models trained on 3T images might demonstrate enhanced performance in tasks requiring high anatomical detail and contrast sensitivity. Conversely, since 1.5T MRI images have lower SNR and contrast, models trained on these images might require more robust preprocessing and data augmentation techniques to achieve similar performance levels. However, training deep learning models exclusively on high-field strength MRI data might limit their generalizability. Therefore, incorporating images from different field strengths into the training set improves the model's ability to generalize across scanners, ultimately enhancing performance on unseen data.

Table 6: Single to multi-scan based MRI acquisition and DL models' performances.

DL Performance	SNR variation	Contrast variation	Resolution variation	Susceptibility to artifact	Higher Learning information	Model training generality	Preprocessing and augmentation	Robustness
Single scanner MRI	✗	✗	✗	✗	✗	✗	✓	✗
Multi-scanner MRI	✓	✓	✓	✓	✓	✓	✗	✓

The age-wise distribution of health controls and CP subjects into eight groups is tabulated in Table 7. Each subject has four MRI scans, including T1-w, T2-w, Flair, and T1-sag (Sag). The

Table 7: The age wise sample distributions for health controls and patients into eight groups.

Subect	G_1	G1_3	G3_5	G5_8	G8_11	G11_13	G13_15	G15_18
Age range	<1	>1:<=2	>2:<=4	>4:<=7	>7:<=10	<10:<=13	>13:<=15	>15
Controls	16	68	59	101	82	49	10	3
CP	52	95	90	54	13	10	3	6

recruited patients' age range is one month to 17 years, with a mean age of 4.86. The underlying models were trained on three MRI machines, including Skyra, GE, and Philips, for dataset collection in the Radiology Department of Shenzhen Children's Hospital in Shenzhen, China. As the collection has been made using different manufactured machines and producing variations in dimensions, the images are transformed to have the exact dimensions, including depth (volume), using Scikit. Precisely, all MRI images were aligned to a size of $320\times320\times17\times1$, with the corresponding height (H), width (W), volume/depth (D), and channel (C). The data set is collected from 2013.1.1 to 2022.10.31. The following parameters are used in the echo-planar imaging (EPI) process to acquire the MRI data: repetition time (TR) = 2000 ms; echo time (TE) = 30 ms; flip angle = 90 degrees; matrix size = 64×64; 32 axial slices; field of view = 24×24cm2; slice thickness = 3mm and no gap. Structure 3D-MPRAGE; T1 Repetition Time [TR, ms] = 2300 ms, Echo Time [TE, ms] = 2.26; Number of Averages =1.0, Slice Thickness =1.0mm, Field of View (FOV)=256mm. Our dataset comprises normal and abnormal CP MRI with different contrasts (T1-w, T2-w, T1-sag, Flair, and TWI). All methods were performed per relevant guidelines and regulations. Table 8 illustrates the inclusion and exclusion criteria.

Table 8: The inclusion and exclusion criteria for the underlying subjects.

Subject	Criteria	Description
CP	Inclusion	1. CP diagnosis was confirmed using the international consensus criteria (i.e., a permanent, nonprogressive motor impairment resulting from a perturbation that occurred in the fetal or infant brain that may be associated with a range of comorbidities including, but not limited to, cognitive, visual, auditory or communicative impairments, along with feeding difficulties or epilepsy.<br>2. Complete routine magnetic resonance examination.
	Exclusion	1. With history of other neurological disorders, traumatic brain injuries, and systemic illnesses.<br>2. With metallic or motion artifacts and poor image quality.
Controls	Inclusion	1. Apgar score >=8 at 1min and 7min after birth. Full term and of normal weight.<br>2. Complete routine magnetic resonance examination and structural MRI showed no abnormality.
	Exclusion	1. With history of perinatal asphyxia, intrauterine distress, or any neurological disease, traumatic brain injuries, or systemic illnesses.<br>2. With metallic or motion artifacts and poor image quality.

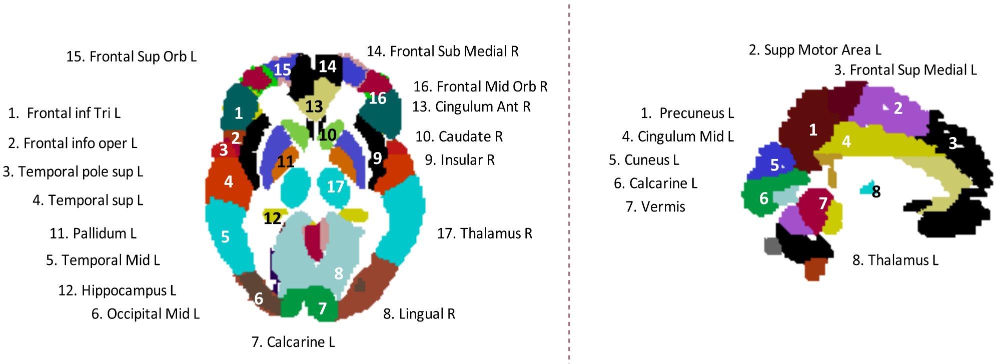

(a) Axial View

(b) Sagittal View

Fig. 3: Demonstration of axial and sagittal views of brain MRI. Different regions are labeled according to their viewed positions.

0.5 Network Settings

The proposed SSeq-DL is trained against the competitor SOTA models. Adam is the optimizer with an initial learning rate of 10^{-5}, $\beta_1 = 0.9$ and $\beta_2 = 0.999$. All the proposed and SOTA ([8–10, 18, 19, 22, 40, 42, 50, 59]) models are trained for 1000 epochs, and after 50 epochs, the learning rate drops by ten decimals. Among 716 subjects, we split the training and testing sets into 615 and 101, respectively. The testing subject consists of 42 patients and 59 controls. Each network has been trained for each single to multi-sequence MRI for fivefold cross-validations. It is preferred to evaluate the models' performance via a confusion matrix. Therefore, we employ a confusion matrix and area under the curve (AUC) to evaluate the models' performance. Furthermore, we visualize the learned filters to distinguish the trends between CP and health controls. The list of MRI slices with covered regions are described in Supplementary Section.

0.6 Results on Single Contrast MRI

This part of the experiment aims to select an optimal SS-MRI-based DL architecture and suitable SS-MRI for CP identification. Therefore, seven DL models are trained on the four SS-MRIs (Table 9). Each DL model has a distinction in their network structure and runs for fivefold cross-validation (Figure 1 and main Table 2. From the training statistics (Table 9), each scan group is composed of numerical results for the seven DL models, where the detailed architectures of the proposed DL models are illustrated in main Table 2. The underlying seven DL architectures' trained results as the area under the curve (AUC) are plotted (main Figure 3), among T2-w was found with higher cumulative AUC score compared to the counterpart SS-MRIs; however, T1-w falls in the second optimal position for CP identification.

The suitability of the T2-w scan for CP prediction is further elaborated and verified using confusion metrics with accuracies ranging from 87.25% to 90.19% (Table 9). On the contrary, the Sag scan was observed to have poor performance. Furthermore, in network architecture-wise selection, Model-6's performance was robust throughout the training on the four SS-MRI scans. It can be deduced from the statistical results that Model-6 is more appropriate while training on SC scans (88.23% using Sag -to 89.21% using T2-w). A few of the models, including Model-3 (89.21% using T2-w), Model-4 (90.19% using T2-w), and Model-7 (90.19%, 89.19%, and 88.23% using T2-w, Flair, and Sag), show slightly better performance; however, poor performance is seen for the rest of the scans (Flair, T1-w, and Sag). Thus, the network structure of Model-6 is employed for CP identification and named SSeq-DL in this study.

Table 9: The SS-MRI-based CP prediction is shown in terms of specificity, sensitivity, and accuracy. The testing split comprises 101 subjects: 59 are health controls, and 42 are health patients. The results of the seven DL models underlying four SSeq-DLs, with significant results in bold, are illustrated. Cumulatively, Model-6 as SSeq-DL was witnessed as robust for CP identification.

Scan	Model	TP	FP	TN	FN	Specificity	Sensitivity	PPV	NPV	F_1	Accuracy%
T2-w	Model-1	56	4	33	9	00.8918	0.8615	0.93	0.78	0.8764	87.25
	Model-2	58	2	32	10	0.9411	0.8529	0.9666	0.7619	0.8948	88.32
	Model-3	55	2	36	6	0.8780	0.9016	0.9166	0.8571	0.8896	**89.21**
	**Model-4**	57	3	35	7	0.9210	0.8906	0.9500	0.8333	0.9055	**90.19**
	Model-5	54	6	31	11	0.8378	0.8307	0.9001	0.7380	0.8342	83.33
	Model-6	56	4	35	7	0.8974	0.8889	0.9334	0.8334	0.8931	**89.21**
	**Model-7**	56	4	36	6	0.9001	0.9032	0.9334	0.8571	0.9016	**90.19**
Flair	Model-1	48	7	30	7	0.8108	0.8772	0.8727	0.8108	0.8406	84.78
	Model-2	56	4	34	8	0.8947	0.8950	0.9334	0.8095	0.8847	82.23
	Model-3	50	10	29	13	0.7435	0.7936	0.8334	0.6904	0.7678	77.45
	Model-4	51	9	38	4	08085	0.9272	0.8501	0.9045	0.8638	87.25
	Model-5	53	7	31	11	0.8157	0.8182	0.8833	0.7380	0.8219	82.35
	**Model-6**	55	5	36	6	0.8780	0.9016	0.9166	0.8571	0.8896	**89.21**
	**Model-7**	54	6	38	4	0.8636	0.9310	0.9001	0.9047	0.8960	**89.19**
T1-w	Model-1	57	3	29	13	0.9082	0.8142	0.9501	0.6905	0.8578	81.74
	Model-2	57	3	29	13	0.9062	0.8142	0.9501	0.6904	0.8578	84.31
	Model-3	56	6	33	9	0.8918	0.8615	0.9334	0.7857	0.8764	87.25
	Model-4	51	9	38	4	0.8085	0.9272	0.8501	0.9047	0.8638	87.25
	Model-5	50	10	38	4	0.9016	0.9259	0.8034	0.9047	0.8535	86.27
	**Model-6**	53	7	37	5	0.8409	0.9137	0.8833	0.8809	0.8758	**88.23**
	**Model-7**	55	5	35	7	0.8750	0.8871	0.9063	0.8334	0.8810	**88.23**
Sagittal	Model-1	48	7	31	6	0.8157	0.8889	0.8727	0.8378	0.8507	85.86
	Model-2	54	6	32	10	0.8421	0.8437	0.9001	0.7619	0.8429	84.31
	Model-3	51	9	36	6	0.8001	0.8947	0.8501	0.8571	0.8447	85.29
	Model-4	55	5	35	9	0.8684	0.8593	0.9066	0.7857	0.8638	86.27
	Model-5	49	11	37	5	0.7708	0.9074	0.8166	0.8809	0.8335	84.30
	**Model-6**	53	7	37	5	0.8409	0.9137	0.8833	0.8809	0.8758	**88.23**
	Model-7	49	11	37	5	0.7708	0.9074	0.8166	0.8809	0.8335	84.31

True positive (TP), False positive (FP), True negative (TN), False negative (FG), Positive predictive value (PPV), negative predictive value (NPV).

0.7 Results on CP Identification Using MS-MRI based Learning

Since MRI can offer images of an object with complementary features, the can be shared between inter-scans in order to learn lesion associated regions. Similarly, MS-MRI images highlight different tissues using different echo and repetition times. Therefore, our employed architectures utilizes both the shared information and inter-sequence differences for association to CP. The Parallel computing unit retains features from both received MRIs into the middle and fuses into a pool of salient features for CP identification. The joint adventure of also discards unassociated voxel information to avoid overfitting and feature saturation. Thus, our experiments are held on the possible combination of MS (Figure S-4) and chosen to train six different DL modalities (Table M-2).

Among the joint adventures of MC-based learning, the fusion of T1-w $\oplus$ Flair and T2-w $\oplus$ Flair showed significantly high results for CP prediction. Furthermore, the fivefold cross-validations for each significant model are shown in Table M-7.The average accuracy for both models, such as T1-w $\oplus$ Flair and T2-w $\oplus$ Flair, are reached to 98.43% and 98.23%. In addition to accuracy as evaluation metrics, confusion matrix, specificity, sensitivity, PPV, and NPV values as evaluation metrics are also significant. Therefore, it deduces that the joint form of a particular MS-MRI increases the accuracy of CP compared to SS-MRI. Similarly, the misclassification of CP from MRI in clinical practices can be reduced to a low level. However, the joint adventure of sagittal and other MRI scans resulted in poor performance. As MS-MRI-based learning receives two scans via a partial siamese unit, the poor performance for the rest of the couplings may be caused by unmatching anatomical structures between the received contrasts. Overall, MS-MRI-based learning outperformed SS-MRI-based learning. However, the learned features will inevitably be exploited further to explain clinical practices logically. For this purpose, the following section analyzes the internal visuals influenced by the incorporated fusion strategies and attention mechanisms.

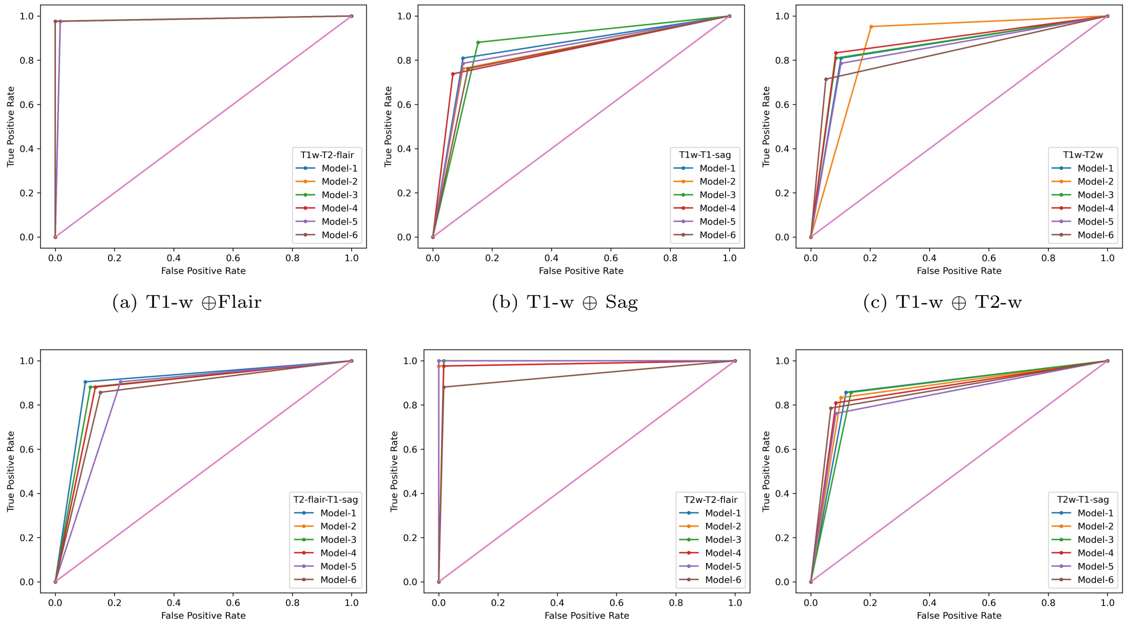

(a) T1-w ⊕Flair (b) T1-w ⊕ Sag (c) T1-w ⊕ T2-w

(d) Flair ⊕Sag (e) T2-w ⊕ Flair (f) T2-w ⊕ Sag

Fig. 4: Six different DL models are trained for couplings with details illustrated in Table 10. The ⊕ denotes coupling, where the left-sided scan feeds as the first input and the right-sided scan feeds as the second input to the parallel units of the underlying model. Among the six couplings, the results for T1-w⊕Flair and T2-w⊕Flair show significant performance for the CP estimation. The rest of the results demonstrate a comparatively poor result.

0.8 Incorporation of fusion for coupling

To address the question raised about improvement in CP prediction by involving MS-MRI, we aim to visualize the learned features as depicted in Figure S-5. There are two models, trained on T1-w ⊕ Flair and T2-w ⊕ Flair, that outperformed the counterparts. Various methods exist for capturing and visualizing the weights acquired by the network structure for specific areas of interest. These strategies include interpreting the black-boxlearning characteristics of deep learning models. There are several methods available for this purpose, such as saliency maps, class activation maps, feature maps, tSNE, Oclussion, LIME, gradient integration, guided backpropagation, and layer-wise relevancey. One may use any appropriate and feasible method, taking into account the characteristics of the dataset, size, application, and intended module. However, in this study, we aim to interpret the attentive regions from different levels of the network structure as well as delve into fusion incorporation and influence toward CP estimation.

0.8.1 T1-w⊕Flair

To visualize the learning capability, firstly, we chose the T1-w ⊕ Flair-based DL model, one of the best performed CP prediction architecture (Figure5). Figure S-5 shows the feature maps corresponding to T1-w and Flair in isolated forms. The fused form (⊕) of the parallel unit receives contrasts depicted in the second row of Figure S-5. We chose a random feature map from a list of maps to extrapolate the merging role by fusion of distinct learned features received from parallel computing. Two MRI scans' representations feed to the parallel network unit, which is visualized in Figure S-2(First row). There are 24 feature maps adopted by each network and followed by the attention mechanism. In the second depth level, we employ 48 feature maps proceeded by fusion. The MRI of each subject contains the width, height, depth (slices), and several channels (Equation S-5).In addition, we randomly selected a slice and depicted its 48 features separately

Table 10: MS-MRI-based CP prediction in terms of specificity, sensitivity, and accuracy. The details of Model-1 to Model-6 have been illustrated where each model has different parameters and hyper-parameters (Table M-2).The coupling with bold shows significant results.

Fused Models	Model	TP	FP	TN	FN	Specificity	Sensitivity	PPV	NPV	F_1	Acc
	Model-1	59	1	41	1	0.9741	0.9833	0.9833	0.9761	0.9797	98.03
	Model-2	56	4	38	4	0.9047	0.9333	0.9333	0.9047	0.9188	92.15
**Fusion of T1-w&Flair**	Model-3	57	3	41	1	0.9318	0.9827	0.9500	0.9761	0.9566	96.07
	Model-4	57	3	40	2	0.9302	0.9661	0.9500	0.9523	0.9478	95.09
	Model-5	58	2	41	1	0.9534	0.9830	0.9666	0.9761	0.9680	97.05
	Model-6	60	0	39	3	0.9285	1.00	0.9523	1	0.9629	970.5
	Model-1	55	5	33	9	0.8684	0.8593	0.9166	07857	0.8638	86.27
	Model-2	48	12	40	2	0.7692	0.9600	0.8000	0.9523	0.8540	86.27
Fusion of T1-w&T2-w(tse)	Model-3	55	5	34	8	0.8717	0.8730	0.9166	0.8095	0.8724	87.25
	Model-4	55	5	35	7	0.8750	0.8870	0.9166	0.8334	0.8810	88.23
	Model-5	54	6	33	9	0.8461	0.8571	0.900	0.7857	0.8516	85.29
	Model-6	57	3	30	12	0.9090	0.8560	0.9500	0.7142	0.8656	85.29
	Model-1	57	3	40	2	0.93023	0.96610	0.9500	0.9723	0.9478	95.09
	Model-2	56	4	38	4	0.9047	0.9333	0.9333	0.9523	0.9047	92.15
**Fusion of T2-w&Flair**	Model-3	55	5	40	2	0.8936	1	0.9166	1	0.9438	95.09
	Model-4	59	1	40	2	0.9756	0.9642	0.9833	0.9523	0.9797	98.03
	Model-5	54	6	40	2	0.8695	0.9642	0.9000	0.9523	0.9144	92.15
	Model-6	59	1	37	5	0.9736	0.9218	0.9833	0.8809	0.9470	94.11
	Model-1	54	6	34	8	0.8500	0.8709	0.900	0.8095	0.8603	86.27
	Model-2	54	6	32	10	0.8421	0.8437	0.900	0.7619	0.8429	84.31
Fusion of T1-w&T1-sag	Model-3	51	9	37	5	0.8043	0.9109	0.8500	0.8809	0.8542	86.27
	Model-4	56	4	31	11	0.8857	0.8358	0.9334	0.7381	0.8600	85.29
	Model-5	54	9	33	6	0.7857	0.9000	0.8571	0.8461	0.8389	85.29
	Model-6	53	7	32	10	0.8205	0.8412	0.8833	0.7619	0.8307	83.33
	Model-1	57	3	33	9	0.9166	0.8636	0.9500	0.7857	0.8893	88.23
	Model-2	56	4	30	12	0.8823	0.8235	0.9333	0.7142	0.8519	84.19
Fusion of Flair&T1-sag	Model-3	52	5	37	8	0.8809	0.8666	0.9122	0.8222	0.8737	87.25
	Model-4	55	10	32	5	0.7619	0.0.9166	0.8461	0.8648	0.8321	85.29
	Model-5	47	13	38	4	0.7450	0.9215	0.7833	0.9047	0.8239	83.33
	Model-6	51	6	36	9	0.8571	0.8500	0.8947	0.8000	0.8535	85.29
	Model-1	53	7	36	8	0.8372	0.8983	0.8833	0.8571	0.8666	87.25
	Model-2	54	6	35	7	0.8536	0.8852	0.900	0.8333	0.8691	87.25
Fusion of T2-w(tse)&T1-sag	Model-3	52	6	36	8	0.8571	0.8666	0.8965	0.8181	0.8618	86.27
	Model-4	52	6	36	8	0.8571	0.8666	0.8965	0.8181	0.8618	86.27
	Model-5	55	5	32	10	0.8648	0.8461	0.9166	0.7619	0.7619	85.29
	Model-6	56	4	33	9	0.8918	0.8615	0.9333	0.7857	0.8764	78.25

Positive Predictive Value (PPV), Negative Predictive Value (NPV)

and together, as seen in the first and second row of Figure S-5. Essentially, both rows have an equal number of filters, such as 48. For illustration, we highlighted the feature map with the number 28 pointed out with the tail of the arrows. Both filters learn distinct features as the inputs are distinct in the white-matters and gray-matters ratio. Therefore, their joint representation (second row, slice number 28) retains features learned by both parallel networks. Similarly, employing fusion at early or later levels shows poor performance by not considering the feature extraction at isolation and joint forms. Therefore, the DL model employing fusion at mid-level shows significant CP prediction.

0.8.2 T2-w⊕Flair

As CP prediction is critical concerning healthcare applications, we also validate and extrapolate the improved learning capacity by combining T2-w and Flair (Table M-7). Prior to fusion modeling, the network model concurrently receives T2-w and Flair scans at the parallel (siamese) networks and combines them (⊕) into a single, more complex network structure. The visual representation demonstrates that certainsliceswithin one partial siamese network exhibit contrasting characteristics that are not present in the mirrored unit. As a result, their collaborative learning enhances the potential for transmitting more CP-related characteristics to a deeper level for decision-making. Significantly, only the fusion at the mid-level yielded noteworthy outcomes, whereas poor performances prevailed for fusions at both early and later levels.

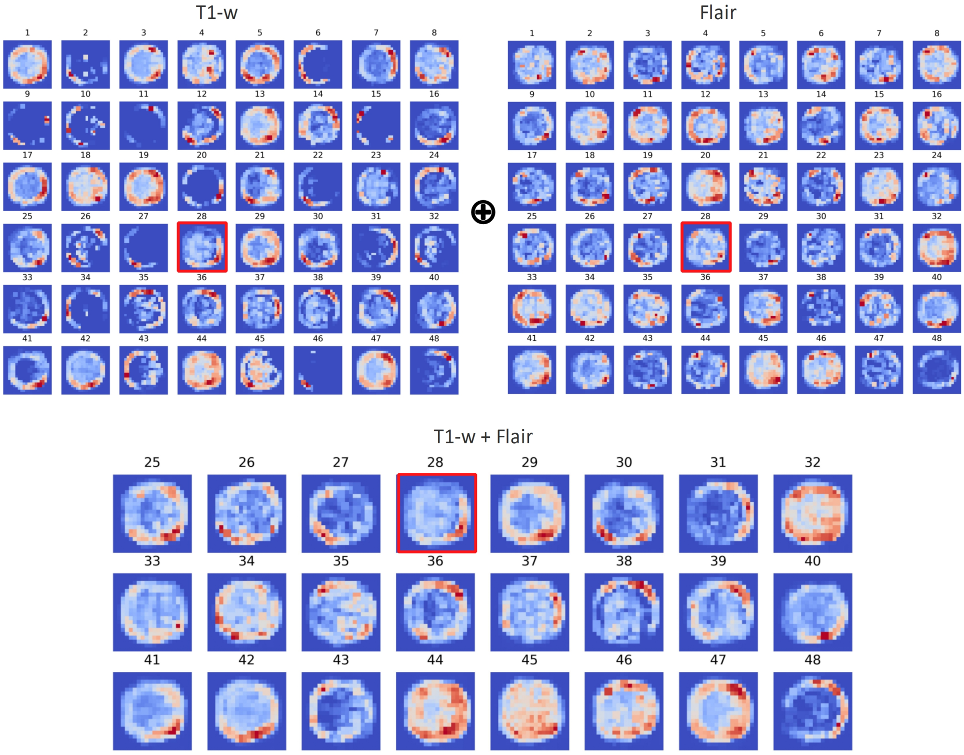

Fig. 5: Visualization of a sample (T1-w ⊕ Flair) from two fusion modalities. The first row depicts the two parallel networks where each network receives correspondingly T1-w and Flair. The second row depicts the joint form of T1-w and Flair, highlighting a slice of interest for only half the number of feature maps.

0.9 Employing attention mechanisms

In addition to fusion modeling, we employ attention mechanisms to emphasize the brain MRI's vulnerable regions and patterns to CP. After making sure the type of attention mechanism (Figure M-1),Table M-5)and the appropriate location (Table 10), two models efficiently performed CP detection from brain MRI (Table M-7).However, in clinical practices, it is inevitable to utilize attention mechanisms to exploit the black box-like notorious attribute attached to DL. There are two types of illustrations to show the fluctuations in learning ability; first, the attention for those DL models outperformed the competitors (Table M-7)and the second one with poor performance (Table 10).

0.9.1 Attention in T1-w⊕Flair based model

The T1-w and Flair-based fused model incorporates an attention mechanism in parallel computation followed by the merging operation. Therefore, we depict the features map from a deep layer of prior and post-employed attention (Figure S-7). There are 24 feature maps shown in rows, each of which applies to all the slices shown in columns. The layers visualization on the left side shows prior attention (Figure S-7(a)) while the right side shows post attention (Figure S-7(b)). The slices in MRI for T1-w and flair are taken from the axial position and aligned to 17 slices for training and evaluation purposes. Notably, the attention mechanism employed in the DL model based on

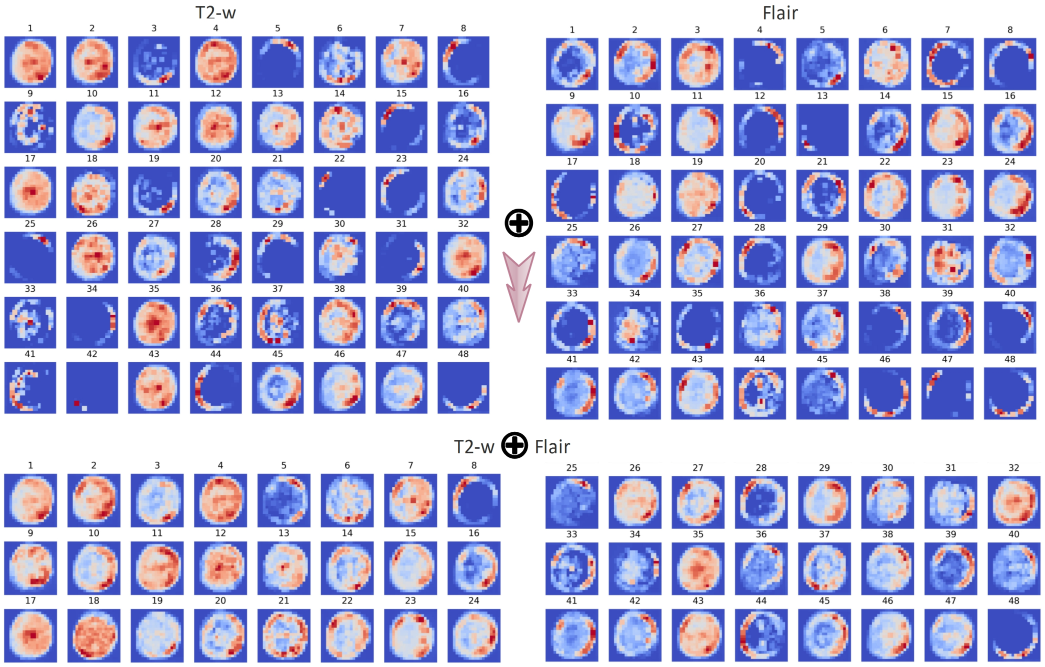

Fig. 6: The visualization of learned weights from the second-best performed DL model incorporating the merging strategies for CP excerpted from Table M-7.In the first row, 48 feature maps are received separately by T2-w and Flair scans. In the second row, the corresponding feature maps merge into a single-line network and pass the salient features to deeper layers to assist in learning CP-related vulnerable regions.

T1-w and Flair mainly focuses on the first and second slices among the list of slices. The rest of the slices receive little attention.

We chose a single slice to visualize more details and passed through the DL model for CP prediction (Figure S-8). The left side (Figure S-8(a)) shows a single slice prior to the attention mechanism while the right side shows post-employed attention (Figure S-8(b)). The model shows higher accuracy, which shows that the early slices are vulnerable to CP identification while using T1-w and Flair as input scans for learning CP-associated patterns. From the feature map visualizations excerpted from the deeper internal structure as learned weights, the DL model trained on T1-w⊕Flair contrasts primarily emphasizes the early slices of each subject. These slices range in the first Scans 1-4 group arranged on slice-based division (Table 1 (First column)). It is noticeable that T1-w⊕Flair deals with the brain MRI from an axial view. Therefore, their slices cover a range of regions in the brain where such regions (shown on the slices) are grouped into four groups. From the depiction of T1-w⊕Flair (Figure S-7), we deduce that the most vulnerable regions include the Insula, Cerebellum, Superior motor area on the left side, and Hippocampus. The detailed description of these regions is listed in the First column of Table 1.

0.10 Attention in T2-w⊕Flair based model

The second DL model outperformed is on T2-w and Flair-based fusion (Table M-7).The architecture of the DL model incorporates the joint adventure of T2-w and Flair contrasts. Both contrasts are received in the partial siamese network unit, where each scan passes through a spatial attention mechanism. There are two possible depictions for T2-w⊕Flair-based modeling, T2-w first fused Flair based (Figure S-9) and Flair first fused T2-w based (Figure S-10). As the model tremendously

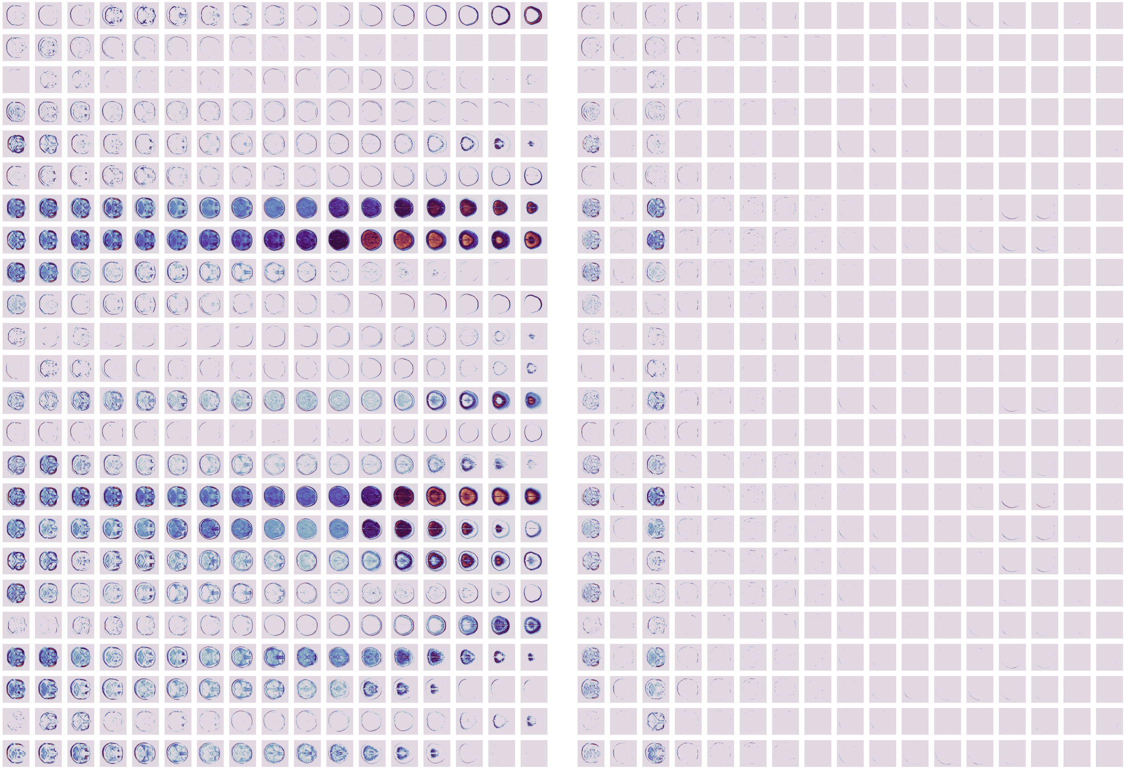

(a) Before employing attention

(b) After employing attention

Fig. 7: Depiction of T1-w and Flair for single subject with all feature maps prior- and post-employing attention.

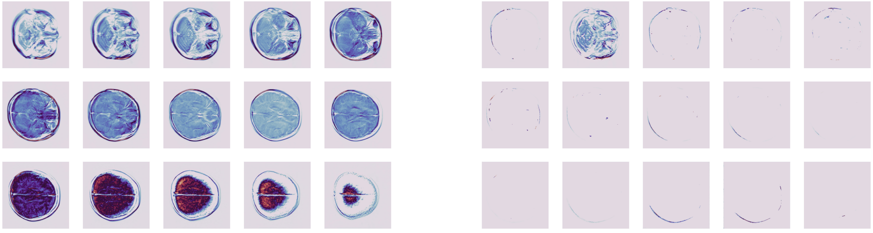

(a) Before applying attention

(b) After applying attention

Fig. 8: A single slice has been selected to visualize more neatly the received attention for CP identification.

performed well, exploiting more internal learning capabilities is necessary. Contrary to T1-w and Flair-based attention modeling, the T2-w⊕Flair-based DL model learns the vulnerable CP-related patterns through various depth slices. The model extracts vulnerable patterns at deeper layers concerning MRI volume corresponding to white and grey matter concentrations. There are two scans received in a parallel computation-based network. The T2-w scan forwards to the network in the first line of partial siamese, followed by the attention mechanism. There are two versions generated, the prior (Figure S-9(a)) and the post attention mechanism based (Figure S-9(b)). The employment of Attention in this experiment selects key features or reduces the learned features into salient ones to minimize the burden on the deeper layers. Similarly, Attention also emphasizes learning different aspects from the brain MRI, starting from inferior to mid and then from

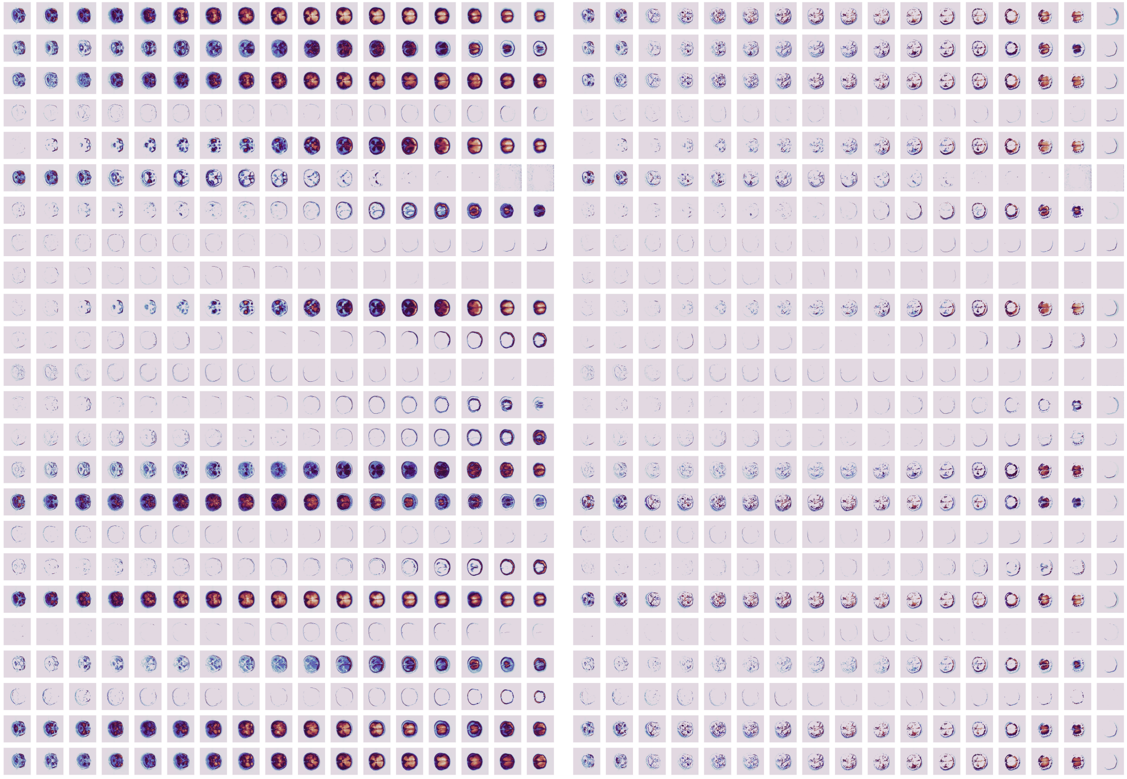

(a) Before employing attention (b) After employing attention

Fig. 9: Visualization of a deep layer from the T2-w and Flair contrasts-based DL model. Both the prior and post-attention mechanism-based depictions are presented. The left side set shows feature maps for T2-w without attention, while the right side demonstrates the effect of employing the attention mechanism. However, a single-slice visualization is shown with more details in Figure S-11.

mid to superior. The influential patterns that the attention mechanism captures include cerebral hemispheres, brain stem, and ipsilateral cerebellar hemisphere. A single slice's detailed and close visualization can be observed in Figure S-11.

Similarly, the Flair sequence for the same subject passes through the second channel of parallel computing (Figure S-10). We visualize both prior (Figure S-10(a)) and post (Figure S-10(b)) employed attention-based feature maps. The DL model learns weights from T2-w scans that differ in CP-related patterns in distinct depths, reflecting varieties of regions in the brain. The vulnerable patterns may lie between the cerebral cortex and cerebellum. It can be noted that the T2-w and Flair scans are acquired from an axial view to capture CP sensitivity better. A closed view can be observed in Figure S-11. In parallel computation, the salient feature maps pass through a fusion stage and forward the merged form to deeper layers for CP prediction. In this way, the model can capture sensitive regions and white-to-grey matters for CP identification. Observing such regions through a naked-eye examination and conventional methods is challenging for clinical doctors and domain experts. In T1-w⊕Flair and T2-w⊕Flair-based models, the typical scan is Flair and achieved 98.19% accuracy. However, the DL model based on isolated Flair contrast achieved 89.10% (Table M-5). From the illustration as mentioned earlier and visual depictions (Figure S-9 and 10), it can be observed that the T2-w⊕Flair based model learns CP associated sequence at distinct depth levels. As both T2-w and Flair sequence view the brain MRI from an axial view, all the axial view-based brain regions are listed in Table 1. Interestingly, the model learning is not limited to the first four slices (Group-1) but considers the complete list of slices. The involvement

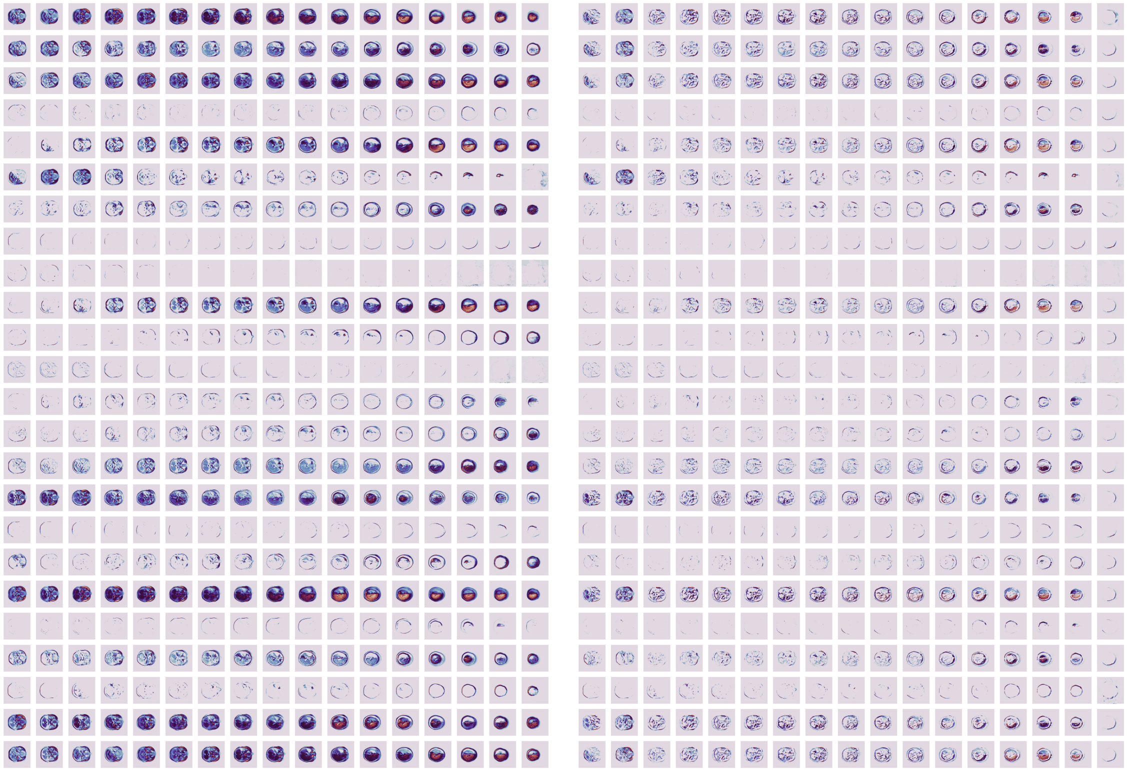

(a) Before employing attention (b) After employing attention

Fig. 10: The T2 and Flair-basedFlair-based fusion model visualization reflected attention effects. Contrary to T2-w first (Figure S-9), this depiction shows the Flair first based attention mechanism.

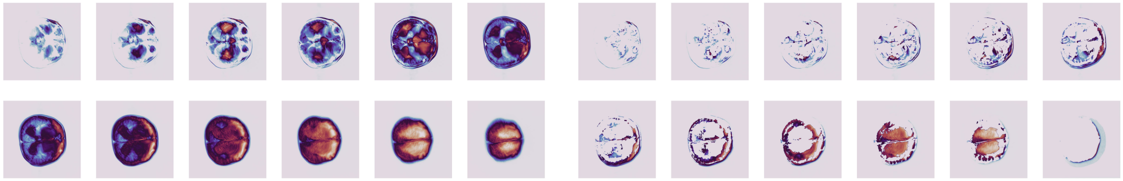

(a) Before employing attention (b) After employing attention

Fig. 11: A single slice employed feature maps are shown to observe the learned patterns regarding CP identification closely.

of Tw-2 enables the model to learn regions outlined throughout the slices obtained from inferior to superior of the head orientation.

0.10.1 Employing attention on Sag based fusion

In addition to the substantial accuracies of T1-w⊕Flair and T2-w⊕Flair-based modeling, we also employ the fusion of T1-sag and other MRI contrasts to exploit the CP association. The models train on T1-w⊕T2-w, T1-w⊕T1-sag, T2-w⊕T1-sag , and on T1-s⊕Flair show poor performance for CP prediction (Figure M-10). In a list of possible joint adventures, the employment of T1-sag with ⊕T1-w and ⊕Flair shows poor results (Table 10). To exploit the internal learned structures of such models, we arrange the T1-sag and Flair-based model's visualizations (Figure S-12 and 13).

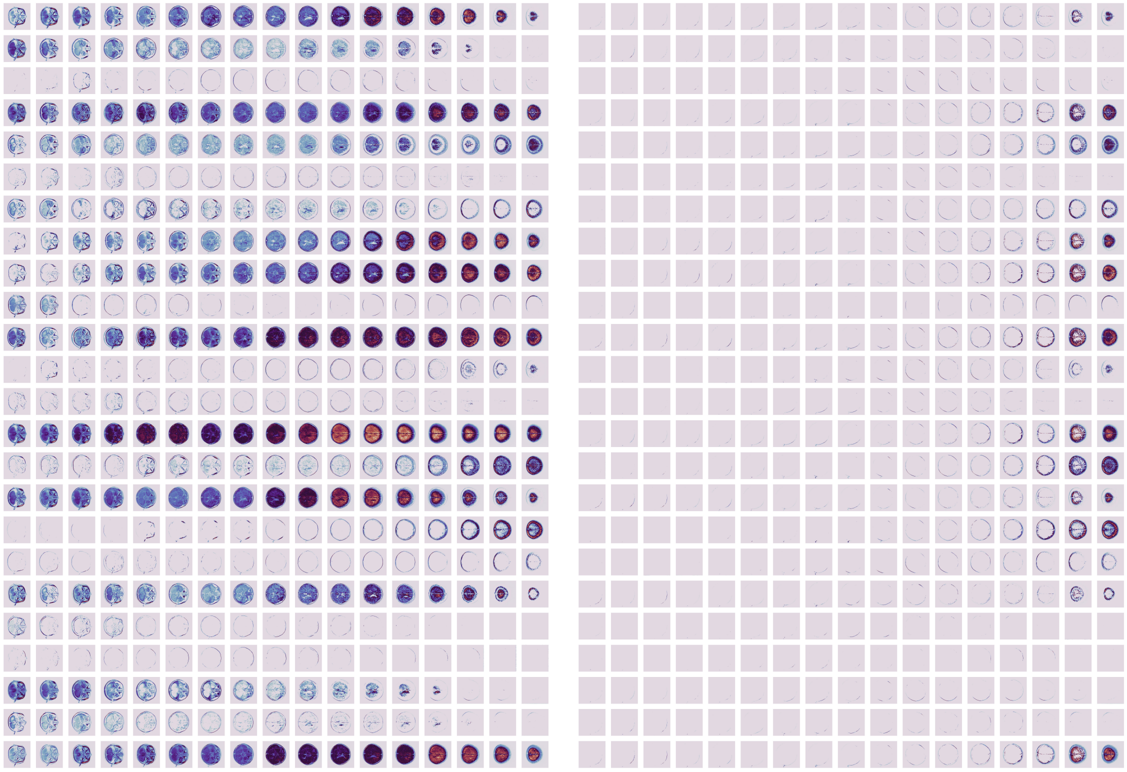

(a) Before employing attention (b) After employing attention

Fig. 12: Visualization of feature maps corresponding to Flair of the underlying subject. The two groups depict the effects on learning network weights pre- and post-employed attention mechanisms. The other parallel receiving scan for the same subject is shown in Figure S-13.

Figure S-12 and 13 depict the corresponding two sets of feature maps where each for prior (Figure S-12 (a)) and post attentions (Figure S-13). In the involvement of a Sag view scan, the DL model emphasizes the last slices of the brain MRI. It deduces that the early and mid-slices in the brain MRI are not sensitive to CP vulnerable features or lesions. Interestingly, the model considers the last layers for both the Flair (Figure S-12) and T1-sag (Figure S-13); however, the Flair scan incorporates varieties of patterns at distinct slices' depth (Figure S-10). As the Sag view-based scan has been considered less sensitive to CP identification, this can be a benchmark for future studies regarding CP-related patterns learning using DL models. Interestingly, we noticed that the DL models involving Sag capture CP-associated patterns from the deeper slices (Figure S-12 and 13). Therefore, the Sag-employed DL model with attention mechanism emphasizes more on the slices range from 14 to 17 (Group-4) (Table 2). The visual depictions from the deeper layers imply that CP is more associated with Sag as a source contrast. The brain tissues concerning the Sag view may include the Temporal, Rolandic, Parietal, Occipital, Angular, Insula, Cerebellum, and Hippocampus (see Fourth column of Table 2 for more details).

0.11 Ablation Study

0.11.1 Single sequence MRI

This part of the experiment aims to select an optimal SS-MRI-based DL architecture and suitable SS-MRI for CP identification. Therefore, seven DL models are trained on the four SS-MRIs (Table 9). Each DL model has a distinction in their network structure and runs for fivefold cross-validation (Figure 1 and Table 3). From the training statistics (Table 9), each scan group is

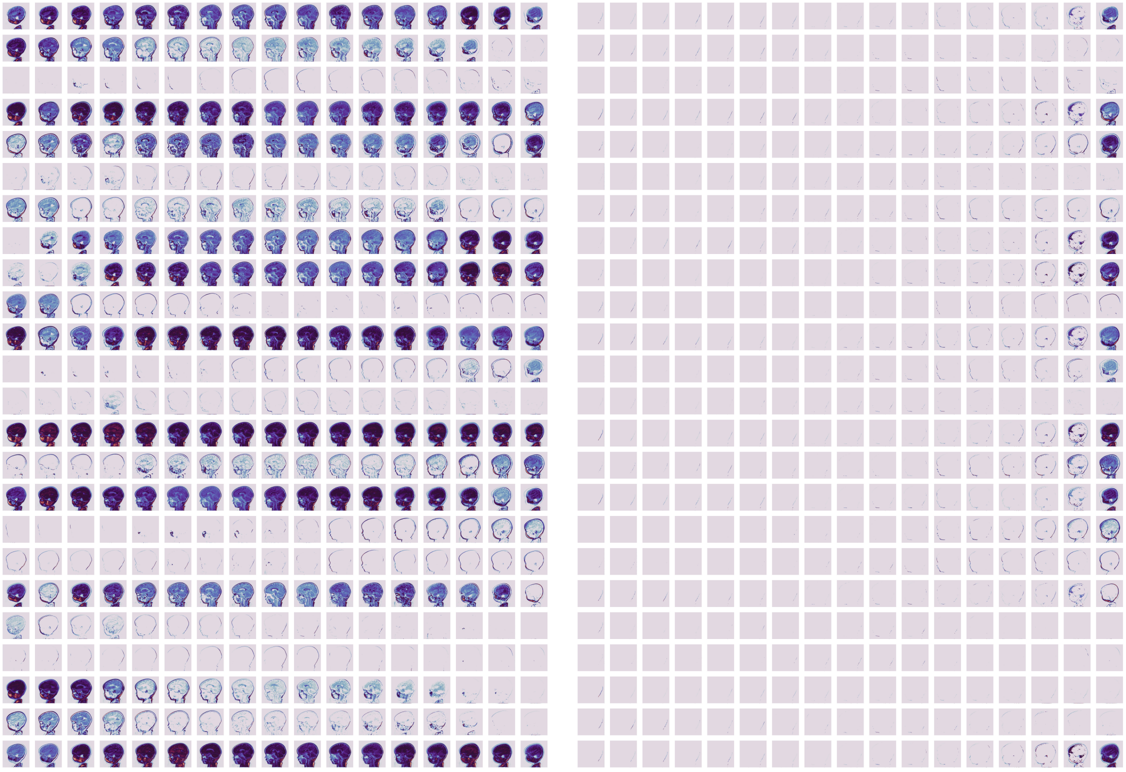

(a) Before employing attention (b) After employing attention

Fig. 13: Similarly to visualization show in Figure S-12, this visualization demonstrate the second MRI scan (T1-sagittal) into the siamese network for the same subject. The pre- (a) and post- (b) attention-employed depictions are shown.

composed of numerical results for the seven DL models, where the detailed architectures of the proposed DL models are illustrated in Table 3. The underlying seven DL architectures' trained results as the area under the curve (AUC) are plotted (main Figure 3), among T2-w was found with higher cumulative AUC score compared to the counterpart SS-MRIs; however, T1-w falls in the second optimal position for CP identification.

The suitability of the T2-w scan for CP prediction is further elaborated and verified using confusion metrics with accuracies ranging from 87.25% to 90.19% (Table 9). On the contrary, the Sag scan was observed to have poor performance. Furthermore, in network architecture-wise selection, Model-6's performance was robust throughout the training on the four SS-MRI scans. It can be deduced from the statistical results that Model-6 is more appropriate while training on SC scans (88.23% using Sag -to 89.21% using T2-w). A few of the models, including Model-3 (89.21% using T2-w), Model-4 (90.19% using T2-w), and Model-7 (90.19%, 89.19%, and 88.23% using T2-w, Flair, and Sag), show slightly better performance; however, poor performance is seen for the rest of the scans (Flair, T1-w, and Sag). Thus, the network structure of Model-6 is employed for CP identification and named SS-DL in this study.

0.11.2 Multi-sequence MRI

The fusion effectiveness at different fusion levels is evaluated under several experiments. In the first model (M-Model-1), the parallel extracted features are fused by employing element-wise multiplication (Table 10). Similarly, M-Model-2 merges the parallel (or partial) networks using element-wise sum, and the rest of the models also use element-wise merging, given the model's performance. Similarly, M-Model-3 employs a spatial attention mechanism and discarded channel

attention based on poor performance. The M-Model-4 and M-Model-5 have dissimilarities regarding SA placement and number of fully connected neurons. Finally, M-Model-6 uses early fusion modelling and prior employing attention mechanism. Among the tabulated models, M-Model-6 is more effective; therefore, the rest of multi-contrast learning carries through M-Model-6. All six models are trained on different combinations of MRI contrasts as shown in Table 10.

0.12 CP Identification from Infancy to Adolescence

CP prediction from infancy to adolescence is critical in radiomics for CP examination. Therefore, this section aims to elaborate on the CP prediction from an early age (a few months) to the Age of 17 years. The testing samples are grouped into five age-wise (AG) groups where the groups fall as: AG_1 ¡=1 year, AG_2 ¿1 and ¡=3, AG_3 ¿3 and ¡=5, AG_4 ¿5 and ¡=10, and AG_5 ¿10 years (main Figure 6(b-c)). Each time, the model trains for five-fold cross-validation represented by CV_1, CV_2, CV_3, CV_4, and CV_5. The first five vertical bars show the five-fold cross-validations, whereas the last bar shows the average of each age-wise group for the cross-validations. For result evaluation, merely T1-w⊕Flair (main Figure 6(b)) and T2-w⊕Flair (main Figure 6(c)) are considered because of their superior performance for CP prediction against all possible couplings. In the evaluation of T1-w⊕Flair (main Figure 6(b)), the overall CP prediction score is lower, whereas the cross-validation statistics show irregular accuracy measures. It extrapolates rapid brain development at an early age compared to adolescence. This trend continues to the later age groups (AG-5), where more consistent CP trends are observed with promising prediction accuracy. Furthermore, the findings of T2-w⊕Flair (main Figure 6(c)) in the early age (AG-1) show a similar trend to that of T1-w⊕Flair (main Figure 6(b)). However, there is a high prediction score for AG-2, which continues smoothly to AG-5. From the models' training findings, the accumulative CP identification score was distinct from the early Age (AG-1) and later Age (AG-5).

0.13 Comparison of SS-DL and SOTA Models

All the underlying SOTA [8–10, 18, 19, 22, 40, 42, 50, 59] are 3D MRI-based architectures with promising prediction results but have never been attempted for CP identification. The proposed SSeq-DL model outperformed the counterpart SOTA models (Table 11). The introduction of SMSeq-DL strives to reduce misclassification (Supplementary Table 4).

Table 11: Comparison of the proposed SSeq-DL and a few selected SOTA models regarding classification metrics.

Network	TP	FP	TN	FN	Specificity	Sensitivity	PPV	NPV	F_1	Accuracy%
[18]	53	7	32	10	0.82	0.84	0.88	0.76	0.83	83
[19]	56	4	33	9	0.89	0.86	0.93	0.78	0.87	78
[8]	6	6	33	9	0.89	0.86	0.93	0.78	0.88	87
[50]	57	3	29	13	0.90	0.81	0.95	0.69	0.86	84
[10]	56	4	34	8	0.89	0.89	0.93	0.81	0.88	82
[59]	7	3	29	13	0.91	0.81	0.95	0.69	0.86	81
[42]	53	7	31	11	0.81	0.82	0.88	0.74	0.82	82
[40]	49	11	37	5	0.77	0.91	0.82	0.88	0.83	84
[9]	50	10	38	4	0.90	0.92	0.80	0.90	0.85	86
[22]	55	5	35	7	0.87	0.89	0.92	0.83	0.88	88
**Our proposed (SSeq-DL)**	57	3	35	7	0.92	0.89	0.95	0.83	0.90	**90**

Positive Predictive Value (PPV), Negative Predictive Value (NPV)

References

[1] Accardo, J., Kammann, H., Hoon Jr, A.H., 2004. Neuroimaging in cerebral palsy. The Journal of pediatrics 145, S19–S27.

[2] Ahmadi, M., O'Neil, M., Fragala-Pinkham, M., Lennon, N., Trost, S., 2018. Machine learning algorithms for activity recognition in ambulant children and adolescents with cerebral palsy. Journal of neuroengineering and rehabilitation 15, 1–9.

[3] Akçakaya, M., Moeller, S., Weingärtner, S., Uğurbil, K., 2019. Scan-specific robust artificial-neural-networks for k-space interpolation (raki) reconstruction: Database-free deep learning for fast imaging. Magnetic resonance in medicine 81, 439–453.

[4] Andermatt, S., Pezold, S., Cattin, P.C., 2018. Automated segmentation of multiple sclerosis lesions using multi-dimensional gated recurrent units, in: Brainlesion: Glioma, Multiple Sclerosis, Stroke and Traumatic Brain Injuries: Third International Workshop, BrainLes 2017, Held in Conjunction with MICCAI 2017, Quebec City, QC, Canada, September 14, 2017, Revised Selected Papers 3, Springer. pp. 31–42.

[5] Bahado-Singh, R.O., Vishweswaraiah, S., Aydas, B., Mishra, N.K., Guda, C., Radhakrishna, U., 2019. Deep learning/artificial intelligence and blood-based dna epigenomic prediction of cerebral palsy. International journal of molecular sciences 20, 2075.

[6] Bertoncelli, C.M., Altamura, P., Vieira, E.R., Bertoncelli, D., Solla, F., 2019. Using artificial intelligence to identify factors associated with autism spectrum disorder in adolescents with cerebral palsy. Neuropediatrics 50, 178–187.

[7] Bertoncelli, C.M., Altamura, P., Vieira, E.R., Iyengar, S.S., Solla, F., Bertoncelli, D., 2020. Predictmed: A logistic regression–based model to predict health conditions in cerebral palsy. Health informatics journal 26, 2105–2118.

[8] Bien, N., Rajpurkar, P., Ball, R.L., Irvin, J., Park, A., Jones, E., Bereket, M., Patel, B.N., Yeom, K.W., Shpanskaya, K., et al., 2018. Deep-learning-assisted diagnosis for knee magnetic resonance imaging: development and retrospective validation of mrnet. PLoS medicine 15, e1002699.

[9] Chefer, H., Gur, S., Wolf, L., 2021. Transformer interpretability beyond attention visualization, in: Proceedings of the IEEE/CVF conference on computer vision and pattern recognition, pp. 782–791.

[10] Chen, S., Ma, K., Zheng, Y., 2019. Med3d: Transfer learning for 3d medical image analysis. arXiv preprint arXiv:1904.00625 .

[11] Cunningham, R., Sánchez, M.B., Butler, P.B., Southgate, M.J., Loram, I.D., 2019. Fully automated image-based estimation of postural point-features in children with cerebral palsy using deep learning. Royal Society open science 6, 191011.

[12] Dale, B.M., Brown, M.A., Semelka, R.C., 2015. MRI: basic principles and applications. John Wiley & Sons.

[13] Doyle, L.W., Crowther, C.A., Middleton, P., Marret, S., 2009. Antenatal magnesium sulfate and neurologic outcome in preterm infants: a systematic review. Obstetrics & Gynecology 113, 1327–1333.

[14] Groos, D., Adde, L., Aubert, S., Boswell, L., De Regnier, R.A., Fjørtoft, T., Gaebler-Spira, D., Haukeland, A., Loennecken, M., Msall, M., et al., 2022. Development and validation of a deep learning method to predict cerebral palsy from spontaneous movements in infants at high risk. JAMA network open 5, e2221325–e2221325.

[15] Gupta, A., Al-Dasuqi, K., Xia, F., Askin, G., Zhao, Y., Delgado, D., Wang, Y., 2017. The use of noncontrast quantitative mri to detect gadolinium-enhancing multiple sclerosis brain

lesions: a systematic review and meta-analysis. American Journal of Neuroradiology 38, 1317–1322.

[16] Herskind, A., Greisen, G., Nielsen, J.B., 2015. Early identification and intervention in cerebral palsy. Developmental Medicine & Child Neurology 57, 29–36.

[17] Himmelmann, K., Horber, V., Sellier, E., De la Cruz, J., Papavasiliou, A., Krägeloh-Mann, I., et al., 2021. Neuroimaging patterns and function in cerebral palsy?application of an mri classification. Frontiers in Neurology , 1889.

[18] Hosseini-Asl, E., Keynton, R., El-Baz, A., 2016. Alzheimer's disease diagnostics by adaptation of 3d convolutional network, in: 2016 IEEE international conference on image processing (ICIP), IEEE. pp. 126–130.

[19] Huang, G., Liu, Z., Van Der Maaten, L., Weinberger, K.Q., 2017. Densely connected convolutional networks, in: Proceedings of the IEEE conference on computer vision and pattern recognition, pp. 4700–4708.

[20] Huff, D.T., Weisman, A.J., Jeraj, R., 2021. Interpretation and visualization techniques for deep learning models in medical imaging. Physics in Medicine & Biology 66, 04TR01.

[21] Illavarason, P., Arokia Renjit, J., Mohan Kumar, P., 2019. Medical diagnosis of cerebral palsy rehabilitation using eye images in machine learning techniques. Journal of medical systems 43, 1–24.

[22] Jang, J., Hwang, D., 2022. M3t: three-dimensional medical image classifier using multi-plane and multi-slice transformer, in: Proceedings of the IEEE/CVF Conference on Computer Vision and Pattern Recognition, pp. 20718–20729.

[23] Jöud, A., Sehlstedt, A., Källén, K., Westbom, L., Rylander, L., 2020. Associations between antenatal and perinatal risk factors and cerebral palsy: a swedish cohort study. BMJ open 10, e038453.

[24] Korzeniewski, S.J., Birbeck, G., DeLano, M.C., Potchen, M.J., Paneth, N., 2008. A systematic review of neuroimaging for cerebral palsy. Journal of child neurology 23, 216–227.

[25] Krägeloh-Mann, I., 2008. Understanding causation of cerebral palsy by using magnetic resonance imaging. paediaTRics and cHild HealTH 18, 399–404.

[26] Krägeloh-Mann, I., Horber, V., 2007. The role of magnetic resonance imaging in elucidating the pathogenesis of cerebral palsy: a systematic review. Developmental Medicine & Child Neurology 49, 144–151.

[27] Krigger, K.W., 2006. Cerebral palsy: an overview. American family physician 73, 91–100.

[28] La Rosa, F., Fartaria, M.J., Kober, T., Richiardi, J., Granziera, C., Thiran, J.P., Cuadra, M.B., 2019. Shallow vs deep learning architectures for white matter lesion segmentation in the early stages of multiple sclerosis, in: Brainlesion: Glioma, Multiple Sclerosis, Stroke and Traumatic Brain Injuries: 4th International Workshop, BrainLes 2018, Held in Conjunction with MICCAI 2018, Granada, Spain, September 16, 2018, Revised Selected Papers, Part I 4, Springer. pp. 142–151.

[29] Lee-Park, J.J., Deshpande, H., Lisinski, J., LaConte, S.M., Ramey, S.L., DeLuca, S.C., 2018. Neuroimaging strategies addressing challenges in using fmri for the children with cerebral palsy .

[30] Li, X., Morgan, P.S., Ashburner, J., Smith, J., Rorden, C., 2016. The first step for neuroimaging data analysis: Dicom to nifti conversion. Journal of neuroscience methods 264, 47–56.

[60] Lima, A.A., Mridha, M.F., Das, S.C., Kabir, M.M., Islam, M.R., Watanobe, Y., 2022. A comprehensive survey on the detection, classification, and challenges of neurological disorders. Biology 11, 469.

[32] MacLennan, A.H., Thompson, S.C., Gecz, J., 2015. Cerebral palsy: causes, pathways, and the role of genetic variants. American journal of obstetrics and gynecology 213, 779–788.

[33] McIntyre, S., Morgan, C., Walker, K., Novak, I., 2011. Cerebral palsy?don't delay. Developmental disabilities research reviews 17, 114–129.

[34] Miller, F., 2005. Cerebral palsy. Springer Science & Business Media.

[35] Miller, F., Bachrach, S.J., 2017. Cerebral palsy: A complete guide for caregiving. JHU Press.

[36] Mohan, P.P., Ramkumar, G., 2024. Experimental evaluation of brain cerebral palsy disease prediction using artificial intelligence assisted learning methodology, in: 2024 Ninth International Conference on Science Technology Engineering and Mathematics (ICONSTEM), IEEE. pp. 1–7.

[37] Moreno-De-Luca, A., Ledbetter, D.H., Martin, C.L., 2012. Genetic insights into the causes and classification of the cerebral palsies. The lancet neurology 11, 283–292.

[38] O'Malley, M.J., Abel, M.F., Damiano, D.L., Vaughan, C.L., 1997. Fuzzy clustering of children with cerebral palsy based on temporal-distance gait parameters. IEEE transactions on rehabilitation engineering 5, 300–309.

[39] Palraj, P., Siddan, G., 2021. Deep learning algorithm for classification of cerebral palsy from functional magnetic resonance imaging (fmri). International Journal of Advanced Computer Science and Applications 12.

[40] Parmar, H.S., Nutter, B., Long, R., Antani, S., Mitra, S., 2020. Deep learning of volumetric 3d cnn for fmri in alzheimer's disease classification, in: Medical Imaging 2020: Biomedical Applications in Molecular, Structural, and Functional Imaging, SPIE. pp. 66–71.

[41] Reid, S.M., Dagia, C.D., Ditchfield, M.R., Carlin, J.B., Meehan, E.M., Reddihough, D.S., 2014. An australian population study of factors associated with mri patterns in cerebral palsy. Developmental Medicine & Child Neurology 56, 178–184.

[42] Roy, S.K., Manna, S., Song, T., Bruzzone, L., 2020. Attention-based adaptive spectral–spatial kernel resnet for hyperspectral image classification. IEEE Transactions on Geoscience and Remote Sensing 59, 7831–7843.

[43] Saha, S., Pagnozzi, A., Bourgeat, P., George, J.M., Bradford, D., Colditz, P.B., Boyd, R.N., Rose, S.E., Fripp, J., Pannek, K., 2020. Predicting motor outcome in preterm infants from very early brain diffusion mri using a deep learning convolutional neural network (cnn) model. Neuroimage 215, 116807.

[44] Sakkos, D., Mccay, K.D., Marcroft, C., Embleton, N.D., Chattopadhyay, S., Ho, E.S., 2021. Identification of abnormal movements in infants: A deep neural network for body part-based prediction of cerebral palsy. IEEE Access 9, 94281–94292.

[45] Szkoda, L., Szopa, A., Kwiecień-Czerwieniec, I., Siwiec, A., Domagalska-Szopa, M., 2023. Body composition in outpatient children with cerebral palsy: A case-control study. International Journal of General Medicine , 281–291.

[46] Tortora, D., Panara, V., Mattei, P., Tartaro, A., Salomone, R., Domizio, S., Cotroneo, A., Caulo, M., 2015. Comparing 3t t1-weighted sequences in identifying hyperintense punctate lesions in preterm neonates. American Journal of Neuroradiology 36, 581–586.

[47] Towsley, K., Shevell, M.I., Dagenais, L., Consortium, R., et al., 2011. Population-based study of neuroimaging findings in children with cerebral palsy. European journal of paediatric neurology 15, 29–35.

[48] Vyas, A.G., Kori, V.K., Rajagopala, S., Patel, K.S., 2013. Etiopathological study on cerebral palsy and its management by shashtika shali pinda sweda and samvardhana ghrita. Ayu 34, 56.

[49] Wang, J., Shen, X., Hu, X., Yang, H., Yin, H., Zhu, X., Gao, H., Wu, Y., Meng, F., 2021. Early detection relationship of cerebral palsy markers using brain structure and general movements in infants born¡ 32 weeks gestational age. Early Human Development 163, 105452.

[50] Yang, C., Rangarajan, A., Ranka, S., 2018. Visual explanations from deep 3d convolutional neural networks for alzheimer's disease classification, in: AMIA annual symposium proceedings, American Medical Informatics Association. p. 1571.

[51] Yang, R., Zuo, H., Han, S., Zhang, X., Zhang, Q., 2021. Computer-aided diagnosis of children with cerebral palsy under deep learning convolutional neural network image segmentation model combined with three-dimensional cranial magnetic resonance imaging. Journal of Healthcare Engineering 2021.

[52] Yoshida, S., Hayakawa, K., Oishi, K., Mori, S., Kanda, T., Yamori, Y., Yoshida, N., Hirota, H., Iwami, M., Okano, S., et al., 2011. Athetotic and spastic cerebral palsy: anatomic characterization based on diffusion-tensor imaging. Radiology 260, 511–520.

[53] Zhang, C.y., Yan, B.f., Mutalifu, N., Fu, Y.w., Shao, J., Wu, J.j., Guan, Q., Biedelehan, S.h., Tong, L.x., Luan, X.p., 2022a. Predicting the brain age of children with cerebral palsy using a two-dimensional convolutional neural networks prediction model without gray and white matter segmentation. Frontiers in Neurology 13.

[54] Zhang, H., Li, H., Dillman, J.R., Parikh, N.A., He, L., 2022b. Multi-contrast mri image synthesis using switchable cycle-consistent generative adversarial networks. Diagnostics 12, 816.

[55] Zhang, J., 2017. Multivariate analysis and machine learning in cerebral palsy research. Frontiers in neurology 8, 715.

[56] Zhang, Y., Ma, Y., 2019. Application of supervised machine learning algorithms in the classification of sagittal gait patterns of cerebral palsy children with spastic diplegia. Computers in biology and medicine 106, 33–39.

[57] Zheng, H., Qu, X., Bai, Z., Liu, Y., Guo, D., Dong, J., Peng, X., Chen, Z., 2017. Multi-contrast brain magnetic resonance image super-resolution using the local weight similarity. BMC medical imaging 17, 1–13.

[58] Zhu, M., Men, Q., Ho, E.S., Leung, H., Shum, H.P., 2021. Interpreting deep learning based cerebral palsy prediction with channel attention, in: 2021 IEEE EMBS International

Conference on Biomedical and Health Informatics (BHI), IEEE. pp. 1–4.

[59] Zunair, H., Rahman, A., Mohammed, N., Cohen, J.P., 2020. Uniformizing techniques to process ct scans with 3d cnns for tuberculosis prediction, in: Predictive Intelligence in Medicine: Third International Workshop, PRIME 2020, Held in Conjunction with MICCAI 2020, Lima, Peru, October 8, 2020, Proceedings 3, Springer. pp. 156–168.

[60] Lima, A.A., Mridha, M.F., Das, S.C., Kabir, M.M., Islam, M.R., Watanobe, Y., 2022. A comprehensive survey on the detection, classification, and challenges of neurological disorders. Biology 11, 469.

[61] Malagi, A.V., Netaji, A., Kumar, V., Baidya Kayal, E., Khare, K., Das, C.J., Calamante, F., Mehndiratta, A., 2022. Ivim–dki for differentiation between prostate cancer and benign prostatic hyperplasia: Comparison of 1.5 t vs. 3 t mri. Magnetic Resonance Materials in Physics, Biology and Medicine 35, 609–620.

[62] Tajima, T., Akai, H., Yasaka, K., Kunimatsu, A., Yoshioka, N., Akahane, M., Ohtomo, K., Abe, O., Kiryu, S., 2023. Comparison of 1.5 t and 3 t magnetic resonance angiography for detecting cerebral aneurysms using deep learning-based computer-assisted detection software. Neuroradiology 65, 1473–1482.

[63] Kushol, R., Parnianpour, P., Wilman, A.H., Kalra, S., Yang, Y.H., 2023. Effects of mri scanner manufacturers in classification tasks with deep learning models. Scientific Reports 13, 16791.
